# The public health co-benefits of strategies consistent with net-zero emissions: a systematic review

**DOI:** 10.1016/S2542-5196(24)00330-9

**Published:** 2025-02-12

**Authors:** Léo Moutet, Paquito Bernard, Rosemary Green, James Milner, Andy Haines, Rémy Slama, Laura Temime, Kévin Jean

**Affiliations:** Modelling, Epidemiology and Surveillance of Health Risks (MESuRS) Laboratory, Conservatoire National des Arts et Métiers, Paris, France; Paris Recherche Santé Environnement Climat (PARSEC), Ecole Normale Supérieure, INSERM, Paris, France; Institut de Recherche en Santé, Environnement et Travail (Irset), Université Rennes, École des Hautes Études en Santé Publique, INSERM, UMR_S, 1085, Rennes, France; Centre on Climate Change and Planetary Health, London School of Hygiene & Tropical Medicine, London, UK; Centre on Climate Change and Planetary Health, London School of Hygiene & Tropical Medicine, London, UK; Department of Public Health, Environments and Society, London School of Hygiene & Tropical Medicine, London, UK; Centre on Climate Change and Planetary Health, London School of Hygiene & Tropical Medicine, London, UK; Department of Public Health, Environments and Society, London School of Hygiene & Tropical Medicine, London, UK; Paris Recherche Santé Environnement Climat (PARSEC), Ecole Normale Supérieure, INSERM, Paris, France; Smile Team, Institut de Biologie de l’Ecole Normale Supérieure (IBENS), École Normale Supérieure, The National Centre for Scientific Research (CNRS), INSERM, Paris, France; Modelling, Epidemiology and Surveillance of Health Risks (MESuRS) Laboratory, Conservatoire National des Arts et Métiers, Paris, France; Modelling, Epidemiology and Surveillance of Health Risks (MESuRS) Laboratory, Conservatoire National des Arts et Métiers, Paris, France; Paris Recherche Santé Environnement Climat (PARSEC), Ecole Normale Supérieure, INSERM, Paris, France; Eco-Evolutionary Mathematics Team, Institut de Biologie de l’Ecole Normale Supérieure (IBENS), École Normale Supérieure, The National Centre for Scientific Research (CNRS), INSERM, Paris, France

## Abstract

Moving towards net-zero emission societies is projected to provide human health co-benefits. However, the magnitude of these co-benefits is poorly documented and might be context specific. Synthesising the evidence on these co-benefits could enhance the engagement of decision makers and populations in climate mitigation actions. We performed database searches of PubMed, Web of Science, and Scopus for studies published between database inception and Jan 1, 2024, identifying 3976 papers. Of these, 58 quantitative studies met our inclusion criteria and were included in this systematic review. These 58 papers explored 125 net-zero emission scenarios and considered various pathways by which climate policies can affect human health. Pathways addressing air quality, physical activity, and dietary changes found substantial health co-benefits, with a median mortality reduction of 1·5%. National or sub-national studies showed that net-zero policies would yield substantial local air quality benefits, independently of the actions taken in neighbouring countries. However, these co-benefits varied with explored emission sector, decarbonisation levers, modelling approach, and location. Studies that included a cost–benefit analysis estimated that monetised benefits outweighed the costs of implementing climate policies. This systematic review highlights the need for a standardised framework to assess and compare health impacts of climate mitigation actions across sectors and confirms that achieving net-zero goals supports far-reaching public health policies.

## Introduction

On Dec 12, 2015, 196 governments adopted the Paris Agreement that aims to reduce anthropogenic greenhouse gas emissions to net zero by 2050 to limit global warming well below 2°C above preindustrial levels.^1^ Resulting nationwide commitments, identified as nationally determined contributions, fall short of addressing these objectives and most of the currently implemented policies do not achieve pledged contributions.^2,3^ In addition to nationally determined contributions, various governmental or non-governmental organisations have been developing roadmaps that outline technical and political solutions for society to attain net-zero emissions (ie, greenhouse gas emissions reduced to the lowest possible level with remaining emissions being offset by natural or artificial carbon sinks).^4,5^ These strategies activate different levers, such as technological innovation to improve energy efficiency and allow decarbonised energy production and political, fiscal, and behavioural instruments to reduce the use of energy and materials, often referred to as demand-side policies.

Many climate mitigation policies are likely to benefit human health by directly and indirectly targeting modifiable environmental and behavioural risks, such as air pollution or diet.^2,6^ Several studies have assessed the health co-benefits arising from either single climate mitigation actions or regional or national multisectorial climate policies.^7,8^ The *Lancet* Pathfinder initiative produced an umbrella review exploring the human health co-benefits of a wide range of specific greenhouse gas mitigation actions.^6^ As yet, no systematic review has explored the health impact of combinations of mitigation actions that aim to achieve net-zero emissions.

Such an appraisal could provide valuable insights to identify specific health pathways, sectors of activity, or levers of decarbonisation that are likely to optimise the co-benefits of climate mitigation actions. Summarising the existing evidence regarding the health co-benefits of pathways to net-zero greenhouse gas emissions is also key to increasing the commitment of people and their governments to climate actions, in a context where implemented or pledged policies fall short of the goals of the Paris Agreement.^9,10^

In this systematic review, we present the current evidence regarding the health co-benefits of prospective net-zero greenhouse gas emission scenarios (hereafter referred to as net-zero scenarios). We compare the predicted health co-benefits across published health impact assessment studies, accounting for various sectors of activity and co-benefit pathways. We also identify the main gaps in knowledge, needs for future research, and provide some recommendations for health impact assessments of prospective net-zero emission scenarios.

## Methods

We conducted a systematic review, following the PRISMA 2020 guidelines.^11^ The PRISMA checklist is available in appendix 2 (pp 5–7). The study protocol was preregistered on June 5, 2023, in PROSPERO (CRD42023429759).

### Search strategy and selection criteria

We searched PubMed, Web of Science, and Scopus for studies published between database inception and Jan 1, 2024. The search query included two mandatory terms, referring to health or mortality on the one side, and to net-zero emissions targets or limited climate change on the other. Health and mortality terms included “health*” OR “mortality” OR “death*”. Net-zero emissions targets or limited climate change terms included “net zero” OR “net-zero” OR “decarboni*” OR “transition scenario” OR “carbon neutrality” OR “Paris Agreement” OR “climate change act” OR “climate change action*” OR “climate change acts” OR “climate change target*” OR “below 2°C” OR “below 1·5°C” OR “limited to 2°C” OR “2°C scenario” OR “2°C trajectory” OR “limited to 1·5°C” OR “1·5°C scenario” OR “1·5°C trajectory”. The detailed search strategy and selection criteria are available in appendix 2 (p 8).

### Screening

Studies identified in the database searches were screened by two independent reviewers (LM and KJ) using the Covidence systematic review software (Veritas, Health Innovation, Melbourne, Australia). A third researcher (LT) resolved any conflicts.

Screening was first carried out based on titles and abstracts, from which only original research pieces were included. At this stage, we only included studies explicitly referring to a greenhouse gas emission objective and assessing quantitative health outcomes or an economic valuation of health impacts. Qualitative studies, reviews, meta-analyses, or opinion pieces were excluded, although we screened meta-analyses and reviews for potential studies to include. 2490 studies were excluded at this screening stage.

In the subsequent full-text assessment, we included studies that relied on a prospective scenario that included socioeconomic or technical choices sufficient to attain net-zero greenhouse gas emissions or to limit climate warming to 1·5°C to 2°C, as called for in the Paris Agreement.^2^ According to the Net Zero Coalition, emissions need to reach net zero by 2050 or shortly after to limit global warming to 1·5°C.^12^ Studies also had to provide quantitative estimates of health impacts or economic assessments of such benefits, and had to explore at least one health co-benefit pathway of mitigation actions.

Co-benefit pathways were defined as the improvement of human public health issues that are not mediated by climate, but would be addressed by climate mitigation policies. Co-benefit pathways included, but were not a priori limited to, air quality improvement, enhanced active travel, and healthy dietary patterns. We considered the reduction of exposure to extreme heat or other climate change impacts as direct benefits of climate mitigation policies, and therefore excluded them from our analyses.

### Data extraction

For all included articles, two authors (LM and PB) independently extracted information on the following characteristics: time period studied, location (eg, worldwide, national, sub-national), emission sectors considered (eg, power generation, transportation, and agriculture, forestry, and other land use [AFOLU]), co-benefit pathways considered (eg, diet, physical activity, air pollution), and assessed health outcome metrics (eg, number of deaths prevented, life-years gained). When available, the disaggregated impacts estimated across different sectors or pathways were extracted. We also retrieved characteristics regarding the modelling methods (eg, demographic hypothesis, models of exposure), health impact assessment approach, and exposure–response function applied (appendix 2 pp 11–14).

For each study (and each scenario assessed when the study assessed several scenarios), we categorised net-zero scenarios based on the major lever of mitigation assumed, using the following in-house categorisation: energy decarbonisation, demand reduction (or sufficiency), health in climate policies, and financial instrument. Further details on categorisation are available in appendix 2 (p 9). Baseline scenarios were also categorised based on their assumptions regarding the evolution of greenhouse gas emissions or utilisation of a reference year (appendix 2 p 15).

### Confidence assessment

Since there is no validated tool to assess methodological bias in health impact assessment studies, we referred to guidelines reported by Hess and colleagues^13^ for modelling and reporting health effects of climate change mitigation actions. Of the 36 modelling or reporting criteria suggested by Hess and colleagues, we retrieved those relevant to our study context and merged them into major topics, resulting in 13 final criteria (appendix 2 p 10).

### Health impact scaling

To compare health impacts across studies, we retrieved and scaled estimates of the number of deaths prevented, life-years gained, or both. When only life-years gained were estimated and if the region of investigation was available in the Global Burden of Disease 2021, life-years gained were converted into premature deaths prevented.^14^ The scaled outcome analysed was the preventable mortality fraction, estimated by the ratio between the number of deaths prevented by a scenario relative to a baseline and the number of deaths projected for the associated location, time, and age range. More details on the scaling calculations are provided in appendix 2 (p 2). Analyses were conducted using R (version 4.2.3) and are available online.

## Results

### Descriptive findings

**Figure 1:**
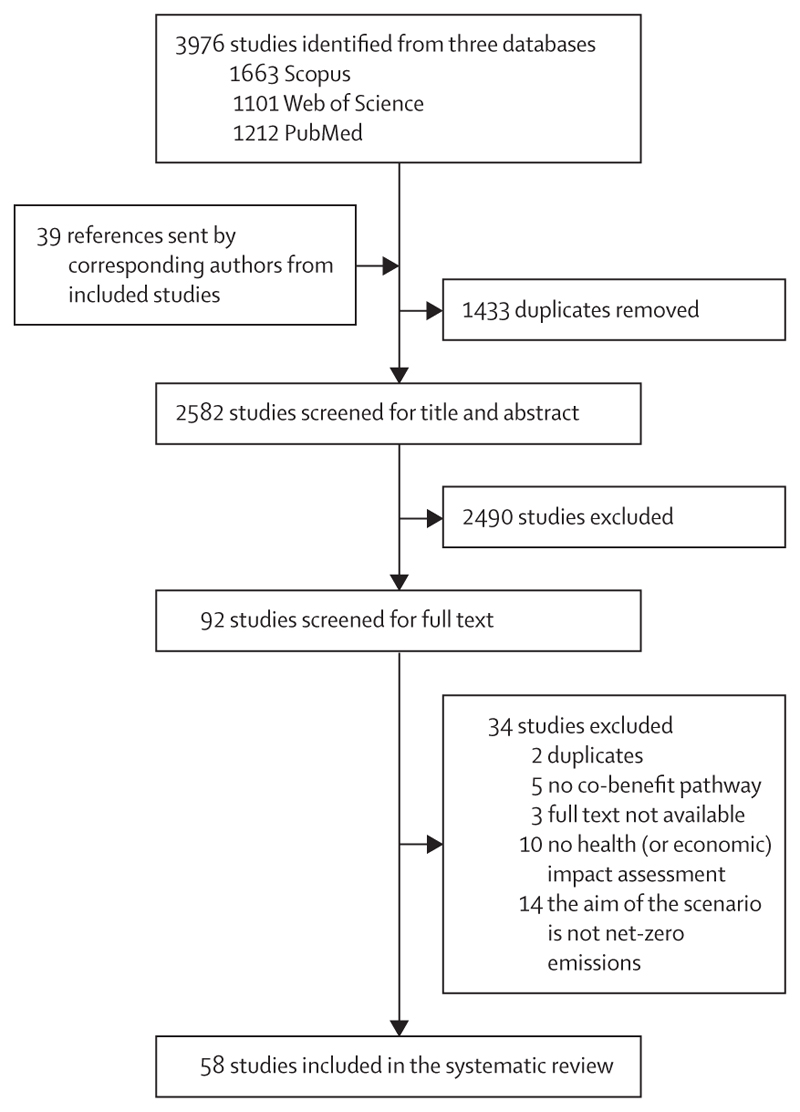
Flow diagram of study selection

We identified 3976 records from the three databases, of which 1433 duplicates were removed (figure 1). All corresponding authors from included studies were contacted in December, 2023, to request potentially relevant unidentified peer-reviewed studies. Of the 2582 abstracts screened, 92 qualified for full-text screening. In the full-text assessment, 34 studies were excluded, mainly because they did not estimate quantitative health metrics (n=10) or because they were not explicitly based on net-zero scenarios (n=14). 58 studies met our inclusion criteria (appendix 2 pp 11–14).

**Figure 2:**
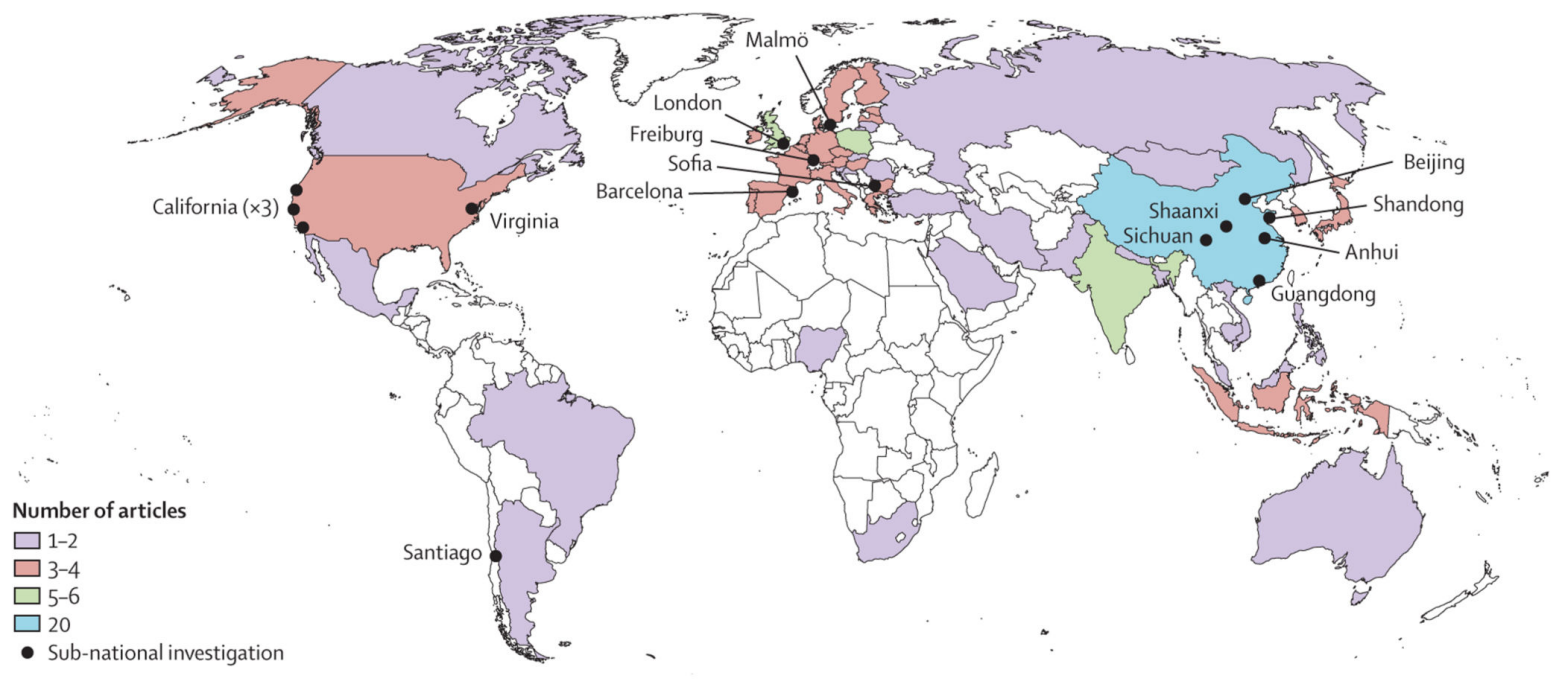
Geographical distribution of studies included in the systematic review The 12 worldwide studies included in the systematic review are not represented on the map.

In addition to 12 worldwide studies,^15–26^ eight studies were conducted on a multinational scale (figure 2) involving between two and 139 countries,^7,8,27–32^ and 25 studies were conducted in single countries. These national assessments focused on northeast Asia,^33–50^ Europe,^51–54^ India,^55,56^ or the USA,^57^ and 13 sub-national studies were conducted in east China,^58–63^ Europe,^64,65^ California (USA),^66–68^ Virginia (USA),^69^ and Santiago (Chile).^70^

**Figure 3:**
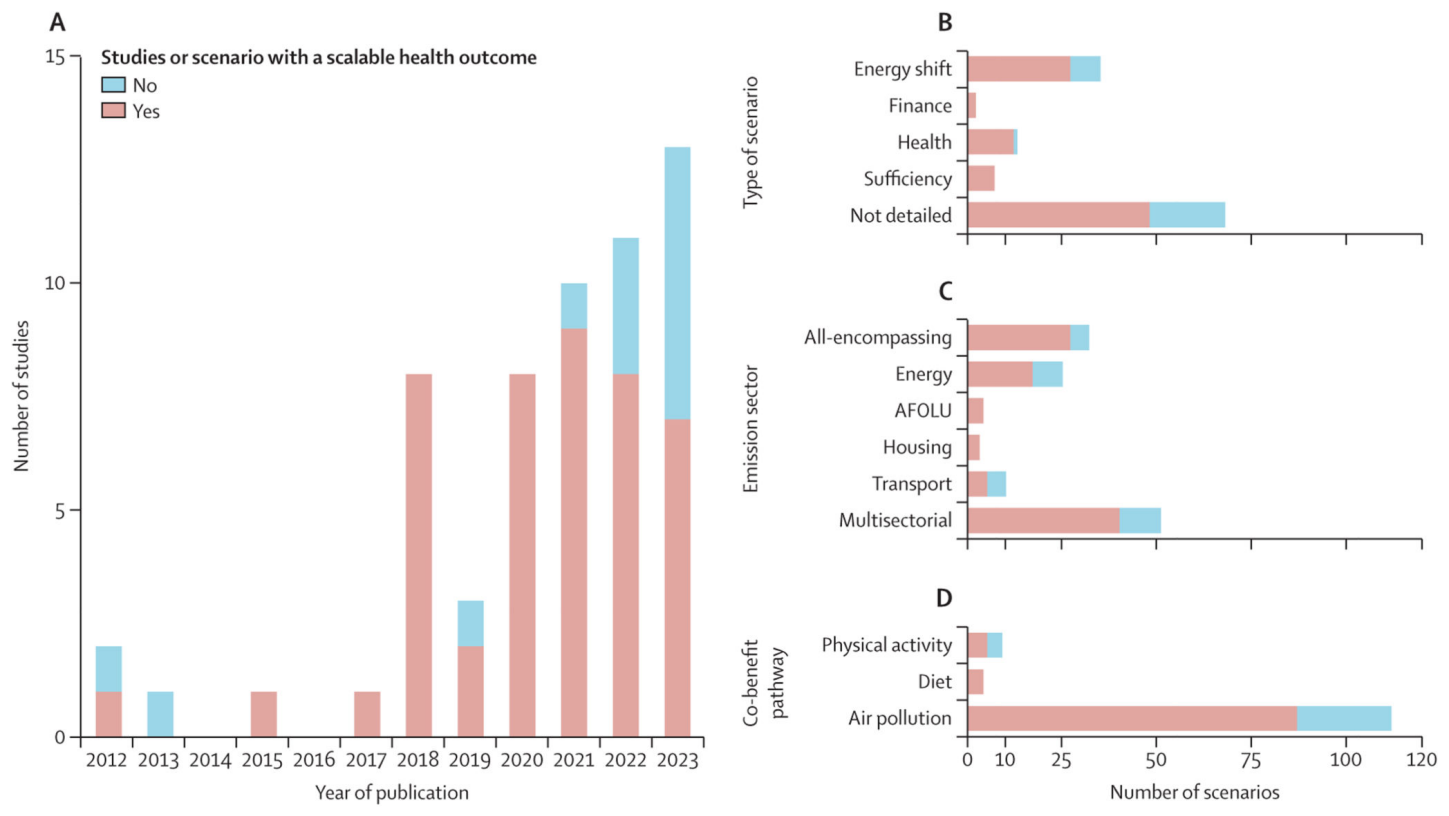
Descriptive analysis of included studies, by publication year (A), type of scenario (B), emission sector (C), and co-benefit pathway studied (D) Some studies included more than one sector in their analyses (ie, multisectorial) and others modelled global anthropogenic emissions (ie, all-encompassing). AFOLU=agriculture, forestry, and other land use.

The main characteristics of included studies are described in figure 3. 53 (91%) of the 58 included studies were published since 2018 (figure 3A).

### Net-zero emission scenarios

14 (24%) of the 58 studies assessed comprehensive scenarios from external prospective net-zero emission plans—ie, developed by a governmental or non-governmental institution. Ten (17%) studies based their scenarios on official nationally determined contributions and 20 (34%) studies relied on the temperature target from the Paris Agreement to estimate subsequent greenhouse gas emissions and air pollution projections. For 14 (24%) studies, the authors developed an in-house scenario (eg, net-zero CO_2_ emission target years for each of the G20 countries) to assess the impacts of various specific measures (appendix 2 pp 3,4).

Of the 125 scenarios presented in the 58 papers, 58 (46%) scenarios provided specific details on the projected levers to achieve net-zero emissions (figure 3B). The main policy lever identified was decarbonisation of the energy sector through the scale-up of technologies, such as carbon capture and storage, renewable energy, electrification, or development of nuclear energy production. Some scenarios aimed specifically at the improvement of human health in a health in all policies approach, most commonly by improving air quality.^7,20,21,23,29,31,40,55,59,64,68,70^ Seven (6%) scenarios relied on demand-side interventions (eg, decreased energy or transport demand).^8,19,33,37,49,54,58^ Four (3%) scenarios relied on financial instruments (eg, carbon taxes or parking pricing)^18,55,64,68^ projected to induce various behavioural shifts (appendix 2 p 15).

### Emission sectors and co-benefit pathways considered

**Figure 4:**
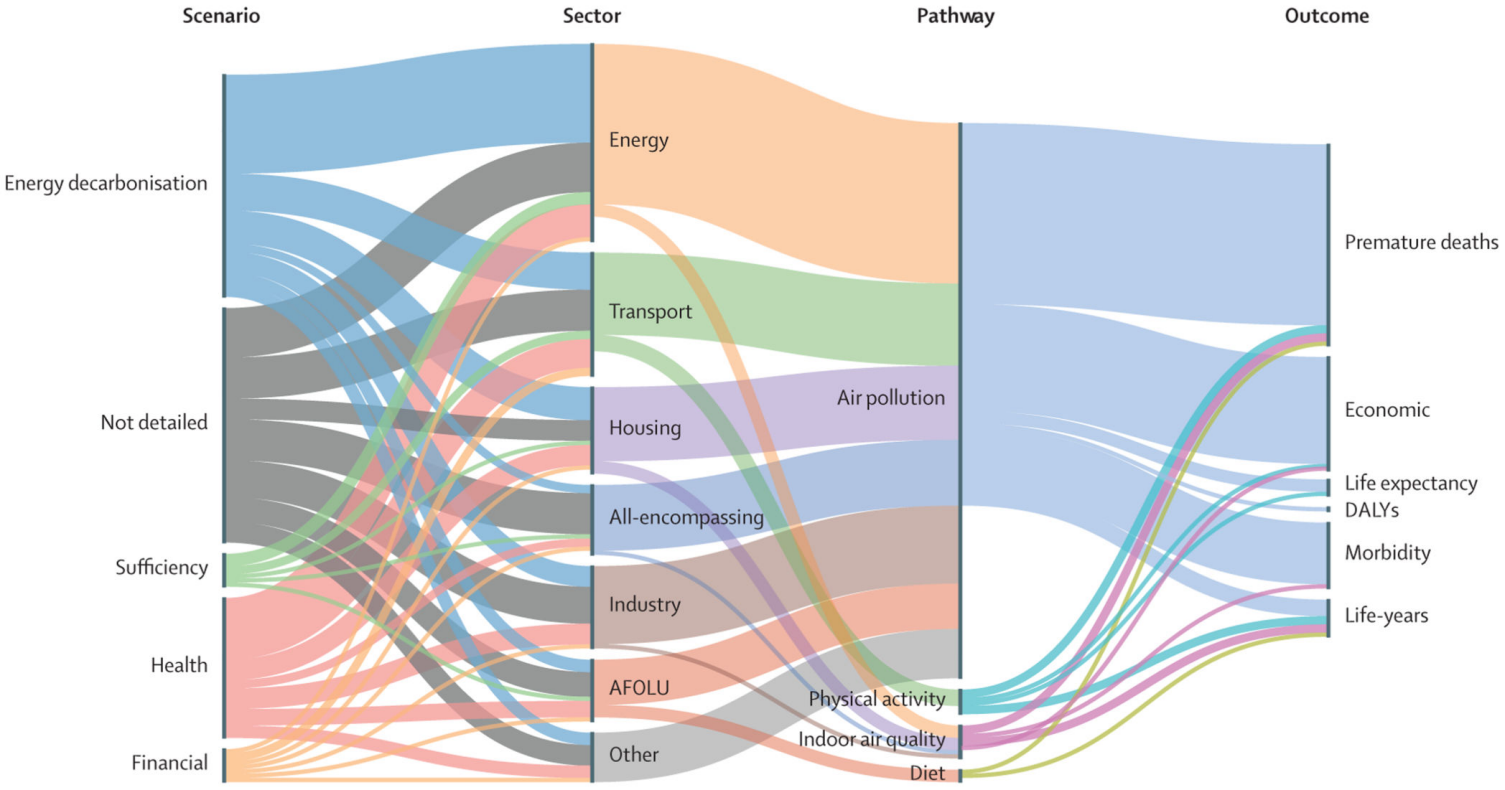
Sankey diagram showing linkage between type of net-zero scenario, emission sector, co-benefit pathway, and health outcome Net-zero scenarios can have links to several emissions sectors, co-benefit pathways, and health outcomes. Air pollution captures ambient air pollution only. Indoor air quality refers to the global quality of the indoor air environment. AFOLU=agriculture, forestry, and other land use. DALYs=disability-adjusted life-years. Several studies focused only on one sector could assess multiple types of scenarios; similarly, several studies only assessing one health pathway could rely on a multisectorial emission model.

For each scenario proposed in the included papers, we explored the emission sector, co-benefit pathway, and health outcome (figure 4).

The emission sectors most frequently studied were energy (n=40), transport (n=27), industry (n=21), housing (n=15), and AFOLU (n=13; figure 3C). 23 (40%) of 58 studies were multisectorial and 14 (24%) studies modelled global anthropogenic emissions (ie, all-encompassing), with 13 (22%) of 58 studies including natural emissions (eg, vegetation fire, dust, sea sprays, and biogenic volatile organic compounds). These studies did not incorporate any specific changes in natural emissions based on the scenarios.

Regarding co-benefit pathways, 56 (97%) of 58 studies assessed health impacts related to air quality, including fine particulate matter or PM_2·5_ (n=53), O_3_ (n=22), SO_2_ (n=4), NO_x_ (n=3), NO_2_ (n=4), and PM_10_ (n=3); five of these studies included indoor exposures to PM_2·5_ (n=5), radon and tobacco smoke (n=2), O_3_ (n=1), increased winter temperature attributable to home energy efficiency (n=1), and mould (n=1). Of the 53 studies including PM_2·5_, 17 (32%) specifically considered black carbon. Five (9%) of the 58 scenarios investigated physical activity enhanced by active travel,^7,8,54^ whereas four (7%) scenarios examined dietary changes with a reduction in red meat consumption (figure 3D).^7,8^ Two (3%) studies combined air pollution, diet, and physical activity,^7,8^ two (3%) studies focused exclusively on physical activity,^54,64^ and one (2%) study focused on indoor air temperature and air quality (ie, PM_2·5_, radon, tobacco smoke, and mould).^65^

### Modelling exposures and outcomes

Various health outcomes were quantified in the 58 studies selected: 46 (79%) estimated the number of premature deaths prevented, four (7%) calculated changes in life expectancy, six (10%) assessed life-years gained, and one (2%) calculated disability-adjusted life-years. Additionally, seven (12%) studies specified morbidity outcomes and 28 (48%) studies conducted an economic assessment. 24 (86%) of these 28 studies used the value of a statistical life-year, five (18%) added a cost of illness assessment, and two (7%) a social cost of carbon assessment. Other studies based their assessment on external costs from the European Commission (n=2),^51,52^ the unit value of health outcome (n=1),^58^ or the cost of conserved energy (n=1).^33^

Several frameworks for modelling exposure were used across included studies to: spatialise air pollution concentrations based on emissions reduction using a single model (eg, GEOS-Chem, Polyphemus) or a model mixture (eg, a combination of WRF-Chem with GAINS); attribute health outcomes to changes in active travel in the population; and attribute health outcomes to changes in dietary patterns in the population.

There were fewer methods to quantify health outcomes, with 44 (76%) of 58 studies using comparative risk assessment methods, 13 (22%) studies relying on lifetable approaches, and one (2%) employing microsimulations.^55^

### Confidence assessment

**Figure 5:**
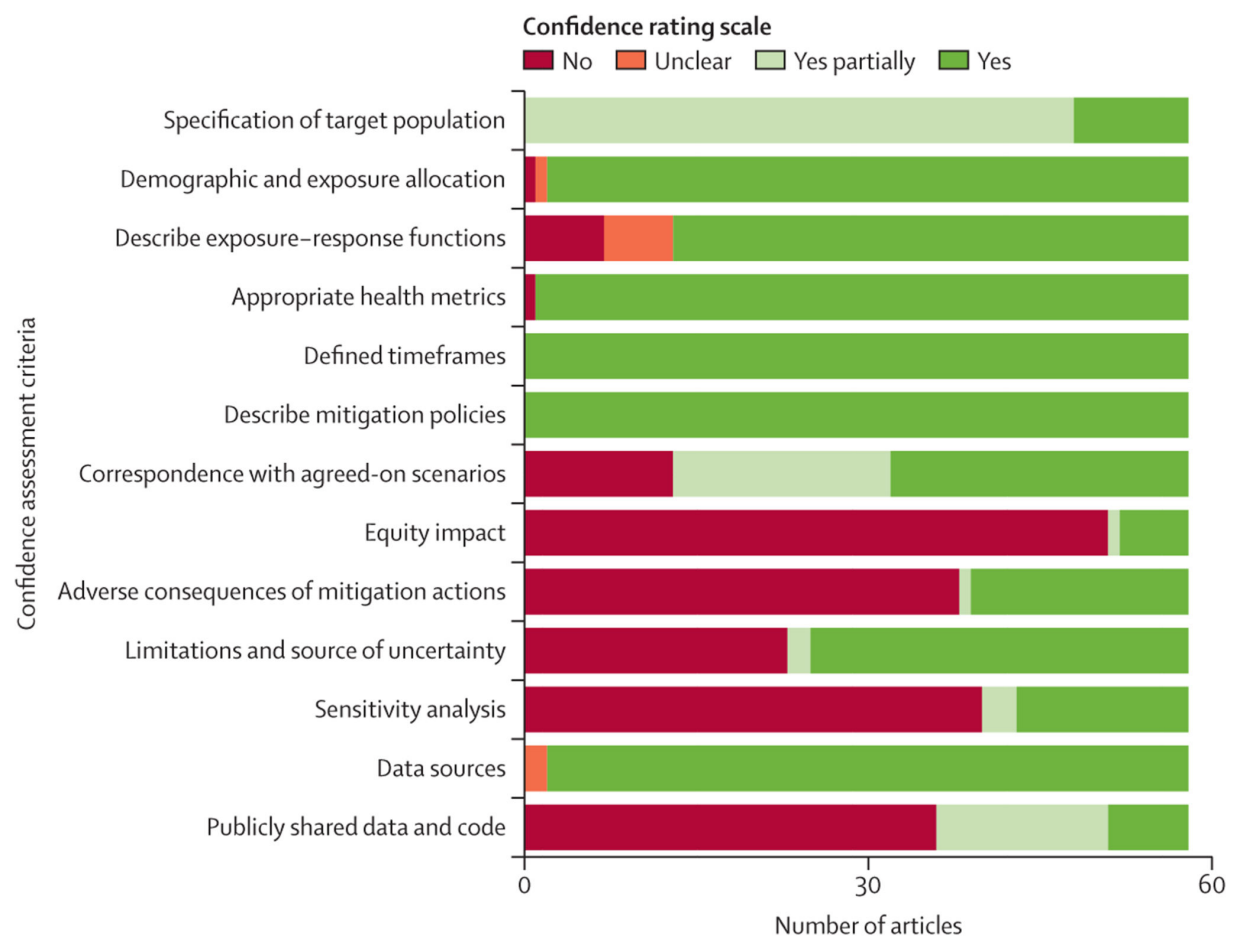
Confidence assessment of included studies per criteria adapted from Hess and colleagues^13^

According to our criteria adapted from Hess and colleagues,^13^ general modelling methods were overall well conducted (including the specification of target population, demographic and exposure allocation, exposure–response functions, health metrics, time-frames, and the description of mitigation policies). The policies, scenarios, and timeframes were well defined, whereas the most overlooked criterion was the evaluation of the equity impacts of policy adoption (figure 5). Discussion of the adverse consequences of mitigation actions, sources of uncertainty, and sensitivity analyses had lower confidence ratings. In addition, very little data and code were publicly available. Detailed results of the confidence assessment by study are available in appendix 2 (pp 11–14).

### Synthesis of the evidence

#### Quantitative health impact

**Figure 6:**
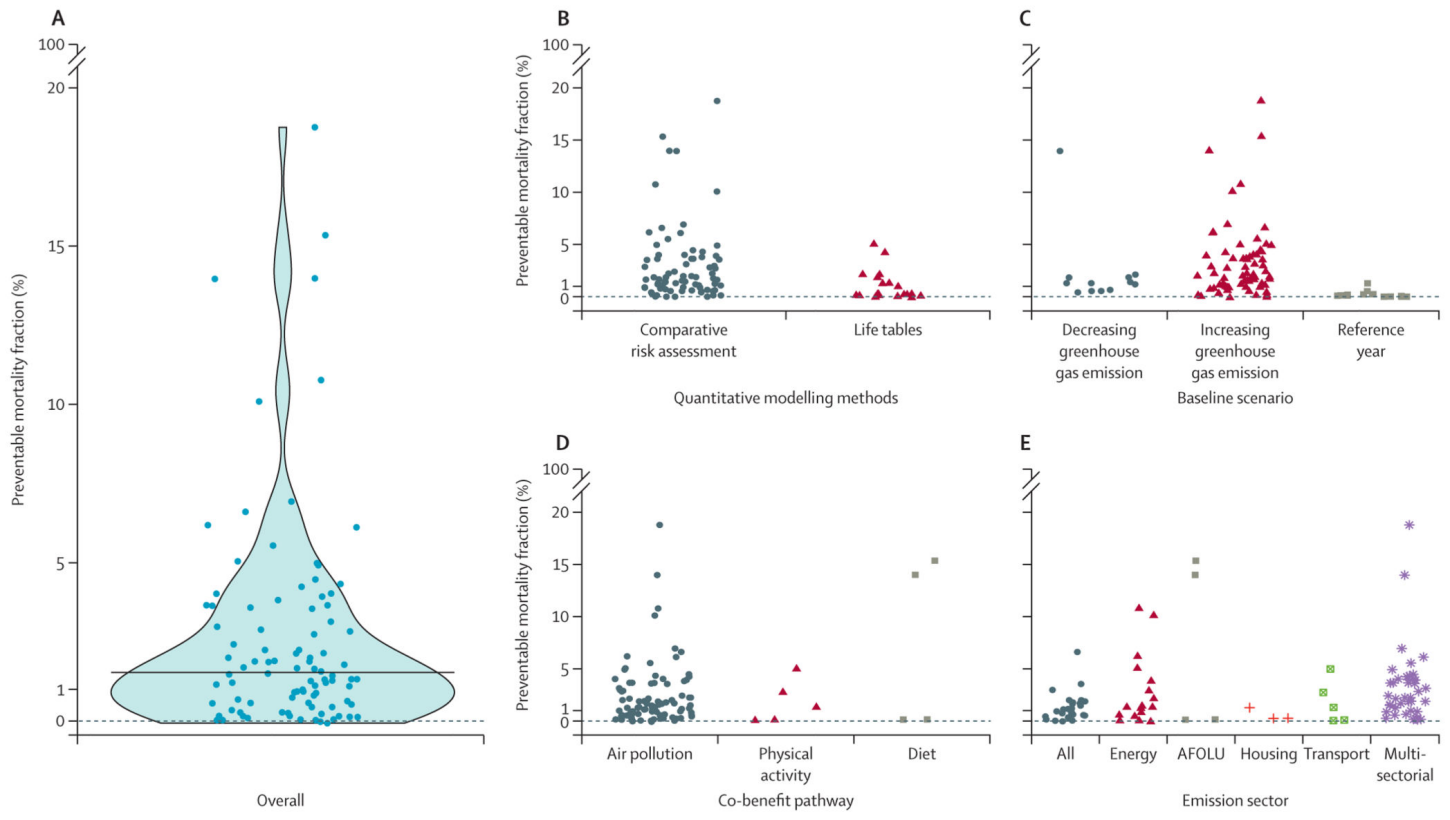
Preventable mortality fraction in various net-zero scenarios (A) All scalable preventable mortality fractions from 96 scenarios across 45 studies with a scalable health outcome. (B) Preventable mortality fraction stratified by quantitative modelling method. (C) Preventable mortality fraction stratified by type of baseline scenario. (D) Preventable mortality fraction stratified by type of co-benefit pathway. (E) Preventable mortality fraction stratified by emission sector. Horizontal bar represents the median value of preventable mortality (ie, 1·5%). AFOLU=agriculture, forestry, and other land use.

We were able to retrieve and scale the preventable mortality fraction of 96 scenarios across 45 studies. Of these scenarios, two (2%; from one study) reported detrimental health impacts (ie, adverse effects on health) in the energy sector (–0*·*09% and –0*·*04% of mortality fraction).^53^ All other scenarios (ie, 94 [98%] of 96) yielded considerable reductions in all-cause mortality, with a median value of 1*·*48% (IQR 0*·*55–3*·*59), and a highest estimated impact of 18*·*74% figure 6A).^47^ The estimated health impacts were on average lower in studies using lifetables (figure 6B) and higher when accounting for increasing greenhouse gas emissions in the baseline scenario (figure 6C); these were also the findings when considering air pollution pathway only (appendix 2 p 16).

Although very few studies assessed the impacts of diet and physical activity pathways, the benefits arising from changing their patterns have the potential to yield substantial health benefits (figure 6D). Modelling emissions from multiple or unique sectors might have provided equivalent health benefits as the use of whole-economy models (figure 6E). We did not identify any single common factor among the scenarios that yielded the greatest health benefits. For the 13 studies that compared the economic benefits arising from health impacts and the implementation costs of the policies, 11 (85%) studies found net benefits and two (15%) found a partial compensation (or a net benefit depending on the country).

#### Health impact across emission sectors and pathways of co-benefits

Most studies focused only on air pollution in association with one or several emission sectors (figure 6D; appendix 2 pp 11–14); resulting health impacts have a wide range, similar to that observed for pathways related to physical activity and diet.

Regarding the most frequently studied air pollutants, PM_2·5_ and O_3_, the sectors associated with the largest health co-benefits were industry, indoor air quality, energy, transport, and AFOLU.^28,44,48,70^ Population density, emission sectors, and baseline levels were important drivers of potential health benefits arising through better air quality.^26,28,39,49,68^ Health co-benefits from decreasing air pollution arose mainly from reduced acute and chronic cardiovascular and respiratory tract diseases.^33,34,50,63^

Increased physical activity also generated substantial public health benefits, comparable to the gains expected from large-scale health prevention interventions.^54^ When comparing different pathways across several countries, Hamilton and colleagues^7^ observed that the attainment of net-zero emissions yielded larger co-benefits through dietary shifts, compared with air pollution reduction or active travel. In terms of health benefits, the ranking of pathways also depended on regional context and the number of mitigation actions modelled.^7,8^

#### Health impact across the typology of net-zero scenarios

Due to a higher potential for reducing air pollution, a scenario that implemented demand reduction policies provided greater health benefits than an energy decarbonisation scenario.^19^ Greater benefits were expected if the energy sector was based on renewable instead of carbon capture and storage technologies.^33^ Scenarios relying on electrification and clean renewable energy in a health in all policies approach can yield four-times more health co-benefits than scenarios featuring combustible renewable fuel.^68^ A city-level study in Beijing, China, found that developing active travel and public transport yielded higher health co-benefits than the electrification of private vehicles (even without accounting for increased physical activity).^59^ Different socioeconomic projections, priorities given, and levels of ambition yielded very different health impacts,^21^ especially for physical activity and diet.^8^

#### Equity impact and regional disparities in net-zero scenarios

Only 6 (10%) of 58 studies explored the distribution of health impacts regarding populations that are socially and economically marginalised. In India, health benefits of net-zero emission scenarios were modelled to be higher for men, individuals living in urban environments, and populations with a high sociodemographic index.^56^ The implementation of integrated climate, air quality, and clean energy access interventions had a synergistic impact, substantially reducing the number of children with stunted growth, particularly those living in the most disadvantaged geographical regions.^55^

Ambitious greenhouse gas reduction efforts in California, USA, provided substantial health co-benefits, especially for residents of communities that are disadvantaged.^66^ In the USA, the enhanced electrification of the transport sector was shown to benefit communities that are disadvantaged more effectively than building electrification.^67^ Accounting for air pollution-related health impacts showed that climate policies have the potential to reduce inequality and increase welfare at several geographical scales, partly because in some regions, the communities that were the most disadvantaged were more exposed.^18,69^ However, even if inequalities were reduced with air quality improvements, they would remain high as long as control measures do not target low-income regions.^22^

Partly due to a high baseline exposure and population density, air pollution co-benefits were the greatest for China and India (appendix 2 p 17).^7,17–19,22–24^ In G20 countries, benefits were mainly attributable to PM_2·5_ emission reduction.^28^ Mitigation policies affecting air pollution emissions had substantial transboundary health impacts, with the transport sector being a major contributor to these benefits.^15,28^ Carbon trading based on historical mitigation rate and low-carbon investment transfer across regions improved the efficiency of global mitigation actions in some contexts.^16^ Disparities in health impacts were also influenced by population ageing, which is expected to increase in the coming years. However, the health co-benefits arising from air pollution mitigation have the potential to offset the effects of population ageing, even for a rapidly ageing country, such as China.^43,45–47,61^

## Discussion

### Review findings

Studies assessing the health impact of scenarios aimed at net-zero emissions show public health co-benefits arising from a range of scenarios, emission sectors, and co-benefit pathways (figure 4). 94 (98%) of 96 scenarios found favourable health impacts that depended on the scenario assumptions, co-benefit pathways, and region of implementation. 48 (50%) of 96 scenarios yielded preventable mortality fractions of over 1·5%, which represents 234 life-years gained per 100 000 individuals (appendix 2 p 18). However, health impacts cannot simply be extrapolated from one setting to another due to heterogeneity in co-benefit pathways, demographic characteristics, modelling methods, and assumptions. 11 (85%) of 13 studies that compared implementation costs with monetised health benefits reported that the costs of net-zero policies would be offset by the economic gains provided by health benefits.

The available evidence mostly focused on three major health pathways: dietary risks have been estimated to be responsible for up to 7 million global deaths annually, air pollution from fossil fuel combustion for 5 million global deaths annually, and physical inactivity for 4 million global deaths annually.^14,71,73^ Similarly to improved dietary patterns, reduced exposure to air pollution would have the potential to yield very important health benefits, especially in high-density and polluted regions.^7^ More comprehensive policies also targeting indoor air quality could yield larger health benefits in some regions.^8^ Active travel policies also have a great potential where low physical activity already induces a high health burden.^7^

Our systematic review identified several sources of variability in the assessed health outcomes. In the reviewed studies, most health outcomes were assessed either by comparative risk assessment methods or lifetable approaches. Comparative risk assessment is a simpler approach, but might overestimate health outcomes because it completely averts a proportion of deaths. Lifetable approaches adopt a more realistic model of deaths over time, as they account for age-specific mortality in the population.^73^ The assumptions regarding the baseline scenario, especially the evolution of greenhouse gas emissions, might affect the magnitude of predicted health outcomes (figure 6C).

Explored scenarios and settings were also highly variable. Energy decarbonisation based on various technologies received the highest attention. However, many net-zero scenarios were not explicit in the transformations assumed to achieve net zero. Despite the high mitigation potential and synergy with wellbeing of demand reduction strategies, these were often marginalised in climate policy and scenarios (appendix 2 p 15), with many studies failing to specify implementation mechanisms.^6,74^ Most studies were performed in high-income regions (appendix 2 p 19) and only a few addressed health inequalities, despite their relevance for public health and environmental justice.^75^

### Implications of the results

Given the long residence time of some greenhouse gases (especially CO_2_) in the atmosphere, accelerated and equitable mitigation actions have the potential to attain net-zero emissions only at mid-term (ie, a decade) to long-term (ie, several decades), depending on the emission sector (2030–35 for AFOLU and 2050 for industry).^3^ Conversely, these same actions have the potential to improve health and wellbeing immediately^2^ by improving cardiovascular, respiratory, and mental health outcomes associated with co-benefit pathways,^76,77^ particularly from air pollution, diet, and physical activity.^8^

Another important feature of the health co-benefits of climate mitigation policies highlighted by this systematic review is their largely unconditional nature. From a climate perspective, mitigation actions must be implemented in most countries and regions to mitigate global warming. As climate benefits are conditional to global coordinated actions, they might be prone to the free-rider problem, where actors do not actively contribute to efforts while expecting to take advantage of collective benefits. Conversely, most of the studies projecting net-zero scenarios reported important health co-benefits while making no specific assumption regarding global coordinated climate actions. In other words, health co-benefits of mitigation policies are largely unconditioned to climate action from other countries or regions, and therefore are likely to be less affected by the free-rider problem. For some pathways (eg, physical activity and diet), the health benefits are restricted to the countries and regions that implement the policies. For air quality, the magnitude of health benefits partly depends on the policies implemented by neighbouring countries,^15,28^ but 34 (97%) of the 35 studies assessing air pollution pathways at a national or sub-national scale revealed that net-zero policies would bring substantial local air quality benefits, independently of the actions taken in neighbouring countries.

Relying on monetary valuation of health impacts, studies have shown that health co-benefits of climate policies have the potential to outweigh the costs of net-zero policies, depending on the region, with India and China showing the largest benefits. The Intergovernmental Panel on Climate Change also reported that the global benefits of climate policies (not accounting for health) exceed the cost of mitigation.^2^ Economic impact assessments anticipate other benefits directly or indirectly affecting human health, such as the net creation of millions of jobs, fewer work days lost, and tens of billions of dollars for labour productivity, crop yield increases, reduced hospital expenditures,^15,27,57,69^ and a more resilient energy system.^20^

### Research gaps

The high heterogeneity of retrieved studies regarding scenarios, emission sectors, co-benefit pathways, and modelling approaches prevented us from drawing conclusions about a clear ranking of co-benefit pathways in terms of potential health impact. In addition, our comparison of health impacts does not account for factors that could potentially lead to differences across studies, particularly due to variations in locations and study populations.

Although our systematic review highlights important health and economic benefits, numerous health impacts remain underestimated. For example, a modal shift to active transportation could provide additional health co-benefits by reducing noise exposure and road travel injuries (if motor vehicles are separated from cyclists and pedestrians).^78^ Health benefits in the transport sector are also expected through improved air quality and mobility independence.^79^ Health impacts related to infectious disease control can also be expected, with various pathways involved.^80^ Included health impact assessments also fail to address mental health impacts, despite evidence suggesting an association between air quality and physical activity with mental health.^76,81^ Adaptation measures that are not accounted for, such as urban green space, also have the potential to yield substantial health benefits.^82^ Incorporating indoor air quality is essential to assess potentially detrimental health impacts associated with poorly ventilated housing.^65^ Lastly, only one study considered the impact of prenatal environmental exposures.^55^

Uncertainties in health impact quantification also result from difficulties in considering multiple variables, such as specific exposure–response functions (eg, across age, sex, or social factors) or the specific distribution of exposures in the studied population. For each mitigation action, there are also potential positive synergistic effects that can be hard to account for in quantitative assessments, such as reduced air pollution emissions along with changes in active travel and dietary patterns. Conversely, extreme climate hazards can restrain cycling behaviours, and health impacts from combined air pollution and heat exposure are exacerbated.^83^ Prospective assessments also assume consistent health-care system efficiency across all scenarios, although higher air pollution and temperature are associated with increased hospital admissions.^84^

Many of the studies and scenarios are from high-income and upper-middle-income regions, where the mitigation efforts are expected to be the greatest, and therefore related societal changes are expected to be important. Whether the magnitude of health co-benefits would be of the same scale in low-income countries remains unclear and will depend on levels of fossil fuel-related air pollution, dietary patterns, and levels of physical activity.^71^ For example, evidence suggests that air pollution reduction (and notably indoor air pollution from cooking stoves) could have a high health co-benefit potential in India.^55,56^ Conversely, one study showed that only modest benefits might be expected in Nigeria from sustainable diet policies.^7^

Evidence on the feasibility and acceptability of implementing assessed actions is scarce. However, known effective interventions include dietary modifications through education, persuasion, and environmental restructuring.^85^ For air pollution, the implementation of effective mitigation strategies requires collaborative actions across multiple stake-holders, including policy makers, civil society, communities, and academia.^86^ In the transport sector, active mobility policies are most effective when integrating safe walking and cycling infrastructure with strong public transport support and educational programmes.^87^ Systems thinking in urban climate policies can enable stakeholders to achieve benefits from multisectorial actions and maximise benefits across climate, health, and urban development.^88^

Finally, we did not investigate grey literature due to methodological issues, and might thus have missed assessments published as reports. Additionally, as our study selection relied on generic search terms, we could have overlooked studies only mentioning disease-specific terms.

### Perspectives and future directions

Here, we make several recommendations for future health impact assessment of net-zero scenarios, inferred from our systematic review.

First, studies should clearly state and justify which mitigation levers are implied by the policy assessed to better estimate the impacts of diverse types of net-zero emission policies.^19,59,64^ Demand-side mitigation policies are essential as they have the potential to induce fundamental lifestyle changes that would support the implementation of sustainable and healthy actions.^74^ Policies and actions must extend beyond technological efficiency improvements to address unsustainable systems that drive high energy and material demands, leading to elevated emissions while neglecting healthy environments.^6^ This aspect is particularly evident in the transport sector, where decarbonisation policies exclusively focused on technological improvements could exacerbate physical inactivity in the population.^89^

As ageing populations can have a substantial impact on estimates,^47^ health impact assessments should prefer lifetable approaches to estimate more accurately health impacts over time; baseline scenarios should include a projection of the studied population to compare the impacts based on the same population pyramid. Prospective health impact assessments of net-zero scenarios should carefully use adapted vulnerability indicators to assess health impacts when possible and should otherwise address inequality impacts qualitatively.^90^ Assessment of energy decarbonisation policies should address energy poverty, which has environmental justice implications.^91^ Our systematic review highlighted that the literature is dominated by results produced in nations that are high emitters of greenhouse gases.

The paucity of code and data sharing by most of the studies presents a barrier to advancing health impact monitoring associated with net-zero scenarios, such as the development of living systematic reviews. Accelerating research and monitoring of health outcomes are essential to provide evidence-based and timely feedback to decision makers.

Although diverse modelling methods could explore wide types of co-benefits and climate actions, a unified framework would be useful to compare the mitigation and co-benefit pathway levers.^13,89^ Such a framework would include description of the exposures, outcomes, pathways, exposure–response function, demographic projections, health impact assessment methods (preferring lifetable approaches and relevant baseline scenarios), and attribution of health outcomes over time.^92^

## Conclusion

Our synthesis of the available evidence suggests that, in high greenhouse gas-emitting countries, achieving net-zero emissions across different sectors would generate large health co-benefits and prevent a considerable fraction of mortality. Therefore, each further delay in implementing transformative changes towards a net-zero society not only increases risks induced by climate change, but also is a missed opportunity to improve human health. Health co-benefits of climate mitigation policies are expected to manifest in the short term, are not conditioned to global coordinated climate action, and can outweigh the costs of mitigation policies, highlighting how health co-benefits can drive impactful mitigation action.

## Supplementary Material

Supplementary Materials
