# Supplementary material for: The public health co-benefits of strategies consistent with net-zero emissions: a systematic review
Source: Lancet Planet Health. Author manuscript; Available in PMC 2025 Dec 1. (PMC7618420; doi:10.1016/S2542-5196(24)00330-9)
Supplement: Supplementary Materials [file EMS211168-supplement-Supplementary_Materials.zip › 1-s2.0-S2542519624003309-mmc2.pdf]

THE LANCET
Planetary Health

Supplementary appendix 2

This appendix formed part of the original submission and has been peer reviewed. We post it as supplied by the authors.

Supplement to: Moutet L, Bernard P, Green R, et al. The public health co-benefits of strategies consistent with net-zero emissions: a systematic review. *Lancet Planet Health* 2025; published online Feb 12. https://doi.org/10.1016/S2542-5196(24)00330-9.

SUPPLEMENTARY MATERIAL

The public health co-benefits of strategies consistent with net-zero emissions: a systematic review of quantitative studies

Table of Contents

Supplementary text 1. Health impact scaling

Supplementary text 2. Elaboration of the scenarios

Table S1. PRISMA checklist

Table S2. Full research equation

Table S3. Confidence assessment grid elaboration

Table S4. Full description of included studies

Figure S1. Typology of net-zero and baseline scenarios

Figure S2. Preventable mortality fraction across net-zero scenarios for air pollution exposure, by health impact assessment methods (A) and choice in the baseline scenario (B)

Figure S3. Health impact of investigations led in China

Figure S4. Life-years gained across net-zero scenarios for total sample

Figure S5. Countries of 1st authors' institution

Supplementary text 1: Health impact scaling

Number of deaths prevented and/or life-years gained were extracted from the included studies. When only life-years gained were estimated, they were converted into deaths prevented in order to compare health impacts across studies.

To do so, we calculated conversion factors from the specific location, age and health outcomes for all-cause mortality reported in the 2021 GBD study (Institute for Health Metrics and Evaluation (IHME), 2024). The conversion factor was linearly extrapolated to the year of interest based on previous years. Mortality projections were based on the number of deaths prevented stated directly in the study or with the GBD tool and the United Nations population prospect 2022, medium fertility variant (https://population.un.org/wpp/Download/Standard/MostUsed/). For sub-national studies in California, (Wang et al., 2020; Zhao et al., 2019; Zhu et al., 2022) we retrieved the mortality rate based on the United States Census Bureau, the number of deaths in 2019 given by the GBD and official population projections. (California Department of Finance. Demographic Research Unit., 2023)

In case only cumulative health impacts over a time range were available, we used the average impact for one theoretical year and the population projections of the median year based on the time-scale of the scenario assessed

Supplementary text 2: Elaboration of the scenarios

- **Comprehensive pathways from a third party** :
  (Schmid et al., 2019) = EU reference scenario 2016: Energy, transport and GHG emissions - trends to 2050 ;
  (Barban et al., 2022) = NegaWatt scenario on transport demand for France ;
  (Milner et al., 2023) = UK Committee on Climate Change, 6th Carbon Budget report ;
  (Williams et al., 2018) = UK Committee on Climate Change, 5th Carbon Budget report ;
  (Shrubsole et al., 2015) = UK Committee on Climate Change, 4th Carbon Budget report ;
  (Hata et al., 2023) = Japan Agency for Natural Resources and Energy, 2022 ;
  (T. Ma et al., 2023) = Synthesis Report 2020 on China's Carbon Neutrality: China's New Growth Pathway ;
  (Shen et al., 2022) = China Energy Interconnection Carbon Neutrality (CEICN) ;
  (Xie et al., 2021) = Based on the government's targets and analysis ;
  (Rafaj et al., 2018) = International Energy Agency's World Energy Outlook 2017 ;
  (Conibear et al., 2022) = International Energy Agency's World Energy Outlook 2018 ;
  (Ortiz et al., 2023) = Virginia Clean Economy Act ;
  (Lu et al., 2022) = Beijing city master plan (2016–35) ;
  (Zyśk et al., 2020) = Energy and emission scenarios of the REFLEX Project ;

- **Based on NDCs** : (Shihui Zhang et al., 2021); (Phillips, 2021); (Cai et al., 2018); (Nawaz et al., 2023b) ; (Polonik et al., 2021)

- **Based on NDCs and adjoins more ambitious objectives** : (Silu Zhang et al., 2021) ; (Qu et al., 2020) ; (Li et al., 2019); (Wang et al., 2022)
  (Hamilton et al., 2021) = Based on NDCs, technical projections (IEA) and hypothesis regarding active transport and dietary patterns;

- **Temperature target only :** (McCollum et al., 2013) ; (Rafaj et al., 2013), (Rafaj et al., 2021) ; (Rauner et al., 2020) ; (Vandyck et al., 2018) ; (H. Wang et al., 2023) ; (Sampedro et al., 2021) ; (Dimitrova et al., 2022) ; (Dimitrova et al., 2021) ; (Shindell et al., 2018)

- **SSP/RCP combination (including temperature targets)** : (X. Ma et al., 2023) ; (Reddington et al., 2023) ; (Shindell et al., 2021) ; (Sampedro et al., 2020) ; (Tang et al., 2022) ; (Y. Wang et al., 2023) ; (Liu et al., 2022) ; (Markandya et al., 2018) ; (Reis et al., 2022) ; (Chen et al., 2018)

- **In-house scenarios made by authors** :
  (Creutzig et al., 2012) = Determined iteratively by stakeholder workshops and interviews in each of the corresponding cities and by comparison to benchmark policies in other cities.
  (Jacobson et al., 2017) = 139 countries reaching full of energy decarbonisation with wind, water and sunlight.
  (Zyśk et al., 2021) = Public electricity and heat production sector in Poland will reach a CO_2 emission reduction of 95% by 2050 compared to the baseline year 1990.
  (Zhu et al., 2022) = Decarbonisation measures that reduce pollutant emissions (reliance on renewable electricity, high electrification of light-duty vehicles buses, and off-road equipment, reductions in demand for petroleum refining…).
  (Yang et al., 2019) = Assumptions of energy conservation and end-of-pipe measures.
  (Xing et al., 2020) = Low carbon policies related to energy conservation for both CO_2 and air pollution in all sectors included.
  (Nawaz et al., 2023a) = Net-zero CO_2 emission target year for each G20 countries.
  (Luo et al., 2023) = Achieves linearly zero emissions by 2050, 2045 or with 20% less carbon emission for each period of 5 years.
  (Lin et al., 2023) = Carbon emission reduction along with forest carbon sink predictions and end-of-pipe control measures.
  (Cheng et al., 2023) = China's CO_2 emission and $PM_{2.5}$ exposure trajectories from 2015 to 2060 and emission peak.
  (Chen et al., 2020) = Decarbonisation based on current industrial and energy structure transformation and development plan.
  (Zhao et al., 2019) = Based on GHG reduction, technology and resource availability and policy trend.
  (Wang et al., 2020) = Sector-based CO_2e emission reduction to determine the changes in infrastructure and technology over time necessary to meet the GHG emissions target.
  (Mo et al., 2022) = Adjoin end-of-pipe control scenarios to the Integrated Policy Model for China (IPAC) model.

Table S1. PRISMA checklist

The PRISMA 2020 statement: an updated guideline for reporting systematic reviews (Page et al., 2021).

Section and Topic	Item #	Checklist item	Location where item is reported
**TITLE**			
Title	1	Identify the report as a systematic review.	Title
**ABSTRACT**			
Abstract	2	See the PRISMA 2020 for Abstracts checklist.	Abstract
**INTRODUCTION**			
Rationale	3	Describe the rationale for the review in the context of existing knowledge.	Introduction, §1
Objectives	4	Provide an explicit statement of the objective(s) or question(s) the review addresses.	Introduction, §1
**METHODS**			
Eligibility criteria	5	Specify the inclusion and exclusion criteria for the review and how studies were grouped for the syntheses.	Methods, §2
Information sources	6	Specify all databases, registers, websites, organisations, reference lists and other sources searched or consulted to identify studies. Specify the date when each source was last searched or consulted.	Methods, §2
Search strategy	7	Present the full search strategies for all databases, registers and websites, including any filters and limits used.	Methods, §2 and Table S2
Selection process	8	Specify the methods used to decide whether a study met the inclusion criteria of the review, including how many reviewers screened each record and each report retrieved, whether they worked independently, and if applicable, details of automation tools used in the process.	Methods, §3
Data collection process	9	Specify the methods used to collect data from reports, including how many reviewers collected data from each report, whether they worked independently, any processes for obtaining or confirming data from study investigators, and if applicable, details of automation tools used in the process.	Methods, §4
Data items	10a	List and define all outcomes for which data were sought. Specify whether all results that were compatible with each outcome domain in each study were sought (e.g. for all measures, time points, analyses), and if not, the methods used to decide which results to collect.	Methods, §5
	10b	List and define all other variables for which data were sought (e.g. participant and intervention characteristics, funding sources). Describe any assumptions made about any missing or unclear information.	Methods, §4
Study risk of bias assessment	11	Specify the methods used to assess risk of bias in the included studies, including details of the tool(s) used, how many reviewers assessed each study and whether they worked independently, and if applicable, details of automation tools used in the process.	Methods, §9
Effect measures	12	Specify for each outcome the effect measure(s) (e.g. risk ratio, mean difference) used in the synthesis or presentation of results.	Methods, §10
Synthesis methods	13a	Describe the processes used to decide which studies were eligible for each synthesis (e.g. tabulating the study intervention characteristics and comparing against the planned groups for each synthesis (item #5)).	Methods, §10; Supplementary text 1
	13b	Describe any methods required to prepare the data for presentation or synthesis, such as handling of missing summary statistics, or data conversions.	
	13c	Describe any methods used to tabulate or visually display results of individual studies and syntheses.	
	13d	Describe any methods used to synthesize results and provide a rationale for the choice(s). If meta-analysis was performed, describe the model(s), method(s) to identify the presence and extent of statistical heterogeneity, and software package(s) used.	
	13e	Describe any methods used to explore possible causes of heterogeneity among study results (e.g. subgroup analysis, meta-regression).	Not applicable
	13f	Describe any sensitivity analyses conducted to assess robustness of the synthesized results.	

Section and Topic	Item #	Checklist item	Location where item is reported
Reporting bias assessment	14	Describe any methods used to assess risk of bias due to missing results in a synthesis (arising from reporting biases).	Not applicable
Certainty assessment	15	Describe any methods used to assess certainty (or confidence) in the body of evidence for an outcome.	Not applicable
**RESULTS**			
Study selection	16a	Describe the results of the search and selection process, from the number of records identified in the search to the number of studies included in the review, ideally using a flow diagram.	Results, §1; Figure 1
	16b	Cite studies that might appear to meet the inclusion criteria, but which were excluded, and explain why they were excluded.	Results, §1; Figure 1
Study characteristics	17	Cite each included study and present its characteristics.	Results, §2; Table S3
Risk of bias in studies	18	Present assessments of risk of bias for each included study.	Table S3
Results of individual studies	19	For all outcomes, present, for each study: (a) summary statistics for each group (where appropriate) and (b) an effect estimate and its precision (e.g. confidence/credible interval), ideally using structured tables or plots.	Results, §4-9; Figure 3
Results of syntheses	20a	For each synthesis, briefly summarise the characteristics and risk of bias among contributing studies.	Table S4
	20b	Present results of all statistical syntheses conducted. If meta-analysis was done, present for each the summary estimate and its precision (e.g. confidence/credible interval) and measures of statistical heterogeneity. If comparing groups, describe the direction of the effect.	Not applicable
	20c	Present results of all investigations of possible causes of heterogeneity among study results.	Results, §4-9; Figure 4
	20d	Present results of all sensitivity analyses conducted to assess the robustness of the synthesized results.	Not applicable
Reporting biases	21	Present assessments of risk of bias due to missing results (arising from reporting biases) for each synthesis assessed.	Not applicable
Certainty of evidence	22	Present assessments of certainty (or confidence) in the body of evidence for each outcome assessed.	Results, §10
**DISCUSSION**			
Discussion	23a	Provide a general interpretation of the results in the context of other evidence.	Discussion, §1
	23b	Discuss any limitations of the evidence included in the review.	Discussion, §8-13
	23c	Discuss any limitations of the review processes used.	Discussion, §8-14
	23d	Discuss implications of the results for practice, policy, and future research.	Discussion, §15-19
**OTHER INFORMATION**			
Registration and protocol	24a	Provide registration information for the review, including register name and registration number, or state that the review was not registered.	Methods, §1
	24b	Indicate where the review protocol can be accessed, or state that a protocol was not prepared.	
	24c	Describe and explain any amendments to information provided at registration or in the protocol.	
Support	25	Describe sources of financial or non-financial support for the review, and the role of the funders or sponsors in the review.	Declaration of interests
Competing interests	26	Declare any competing interests of review authors.	Declaration of interests
Availability of data, code and other materials	27	Report which of the following are publicly available and where they can be found: template data collection forms; data extracted from included studies; data used for all analyses; analytic code; any other materials used in the review.	Declaration of interests

PRISMA 2020 for Abstracts Checklist

Section and Topic	Item #	Checklist item	Reported (Yes/No)
**TITLE**			
Title	1	Identify the report as a systematic review.	Yes
**BACKGROUND**			
Objectives	2	Provide an explicit statement of the main objective(s) or question(s) the review addresses.	Yes
**METHODS**			
Eligibility criteria	3	Specify the inclusion and exclusion criteria for the review.	No*
Information sources	4	Specify the information sources (e.g. databases, registers) used to identify studies and the date when each was last searched.	Yes
Risk of bias	5	Specify the methods used to assess risk of bias in the included studies.	No*
Synthesis of results	6	Specify the methods used to present and synthesise results.	No*
**RESULTS**			
Included studies	7	Give the total number of included studies and participants and summarise relevant characteristics of studies.	Yes
Synthesis of results	8	Present results for main outcomes, preferably indicating the number of included studies and participants for each. If meta-analysis was done, report the summary estimate and confidence/credible interval. If comparing groups, indicate the direction of the effect (i.e. which group is favoured).	Yes
**DISCUSSION**			
Limitations of evidence	9	Provide a brief summary of the limitations of the evidence included in the review (e.g. study risk of bias, inconsistency and imprecision).	No*
Interpretation	10	Provide a general interpretation of the results and important implications.	Yes
**OTHER**			
Funding	11	Specify the primary source of funding for the review.	Yes
Registration	12	Provide the register name and registration number.	Yes

*Not present in the abstract due to word limit but developed in the text.

From: Page MJ, McKenzie JE, Bossuyt PM, Boutron I, Hoffmann TC, Mulrow CD, et al. The PRISMA 2020 statement: an updated guideline for reporting systematic reviews. BMJ 2021;372:n71. doi: 10.1136/bmj.n71

Table S2. Full research equation

PubMed:

**#1**	health*[Title/Abstract] OR mortality[Title/Abstract] OR death*[Title/Abstract]
**#2**	net zero[Title/Abstract] OR net-zero[Title/Abstract] OR decarboni*[Title/Abstract] OR transition scenario[Title/Abstract] OR carbon neutrality[Title/Abstract] OR paris agreement [Title/Abstract] OR climate change act[Title/Abstract] OR climate change action* [Title/Abstract] OR climate change acts[Title/Abstract] OR climate change target* [Title/Abstract] OR below 2°C [Title/Abstract] OR below 1.5°C [Title/Abstract] OR limited to 2°C[Title/Abstract] OR 2°C scenario[Title/Abstract] OR 2°C trajectory[Title/Abstract] OR limited to 1.5°C[Title/Abstract] OR 1.5°C scenario[Title/Abstract] OR 1.5°C trajectory[Title/Abstract]
**#3**	**#1** AND **#2**
**#4**	**#3** Filter: **None**

Web Of Science:

**#1**	TS=(health* OR mortality OR death*)
**#2**	TS=("net zero" OR net-zero OR decarboni* OR "transition scenario" OR "carbon neutrality" OR "paris agreement" OR "climate change act" OR "climate change action*" OR "climate change acts" OR "climate change target*" OR "below 2°C" OR "below 1.5°C" OR "limited to 2°C" OR 2°C scenario OR 2°C trajectory OR limited to 1.5°C OR 1.5°C scenario OR 1.5°C trajectory)
**#3**	**#1** AND **#2**
**#4**	**#3** Filter: **None**

Scopus:

**#1**	TITLE-ABS (health* *OR* mortality *OR* death*)
**#2**	TITLE-ABS ("net zero" OR net-zero OR decarboni* OR "transition scenario" OR "carbon neutrality" OR "paris agreement" OR "climate change act" OR "climate change action*" OR "climate change acts" OR "climate change target*" OR "below 2°C" OR "below 1.5°C " OR "limited to 2°C" OR 2°C scenario OR 2°C trajectory OR limited to 1.5°C OR 1.5°C scenario OR 1.5°C trajectory)
**#3**	**#1** AND **#2**
**#4**	**#3** Filter: **None**

Table S3. Description of the categorisation of net-zero scenarios

**Typology**	**Definition**	**Example of article**	**Example of action**	**Quote from article**
Energy decarbonisation	Reducing GHG emissions arising from energy production or consumption.	(Ortiz et al., 2023)	Electrification	« … *transitioning to fossil fuel-free electric generation…* »
Demand reduction	Reduced demand of consumers due to societal changes.	(Barban et al., 2022)	Demand-side mitigation	« *…the scenario assumes an overall decrease in transportation demand…* »
Health in climate policy	Policies that integrates health considerations into decisions across all economic sectors.	(Dimitrova et al., 2022)	Air quality target	« *…mitigation efforts with targeted air quality and energy access policies…* »
Financial instrument	Financial charges regarding emitting sources or practice.	(Reis et al., 2022)	Carbon pricing	« *…model the achieved TTs [Temperature targets] by imposing a global uniform carbon tax…* »

Table S4. Confidence assessment grid elaboration

**Criteria extracted from guidelines** (Hess et al., 2020)	**Adapted criteria**
Describe mitigation policies and scenarios and their relevant sectors (e.g., finance, energy, transportation, housing, industry, food systems including agriculture).	Describe mitigation policies and baseline scenarios, detailing relevant sectors of emissions, geographic scale and characteristics of the populations.
Specify the geographic scale (international, regional, national, subnational, city) and geographic area of interest.	
Describe populations (size and other characteristics) for model baseline and counterfactual scenarios.	
List and describe counterfactual scenarios, ensuring that these reflect current realities in the absence of strong mitigation policies. As appropriate, list and describe other mitigation policies used in the model.	
If applicable, account for equity and describe socially and economically marginalized populations.	Address equity by describing socially and economically marginalized populations, and evaluate the equity impacts of policy adoption
Describe equity impacts of policy uptake.	
If applicable, account for and describe populations that are most likely to experience adverse consequences or benefit the most.	If applicable, account for and describe populations that are most likely to experience adverse consequences or benefit the most.
Describe how the analyses will account for projected demographic changes	Describe how the analyses will incorporate projected demographic changes and detail the methodology for assigning exposure to mitigation actions, including the proportion of the population exposed over time based on implementation.
Describe how exposure(s) to the mitigation action(s) and related downstream exposure(s) is assigned, including, as appropriate, the proportion of the population that is exposed over time as a function of implementation.	
Describe any potential correspondence between mitigation and counterfactual scenarios with SSPs, RCPs, and/or other globally agreed-upon scenarios, either qualitatively through narrative linkages (e.g., if the mitigation and counterfactual scenarios are characteristic of particular scenarios) or quantitatively (e.g., using specific combinations of RCPs and SSPs with numerical correlates for emissions, demographic shifts, etc.).	Describe any potential correspondence between mitigation and counterfactual scenarios with SSPs, RCPs, and/or other globally agreed-upon scenarios, either qualitatively through narrative linkages (e.g., if the mitigation and counterfactual scenarios are characteristic of particular scenarios) or quantitatively (e.g., using specific combinations of RCPs and SSPs with numerical correlates for emissions, demographic shifts, etc.).
List and describe data sources used for population and demographic projections.	Provide a comprehensive overview of data sources utilized, including those for population, demographic projections and counterfactual scenarios.
Describe data sources used for counterfactual scenarios (for example, emission scenarios).	
Describe and justify sources used for baseline health estimates and demographics (e.g., national vital statistics, the Global Burden of Disease study, etc.).	
Specify and justify the baseline year for data sources used in the model.	Specify the baseline year for data sources used in the model and define time frame and projected time horizons
Clearly define and justify time frames and projected time horizons.	
Describe and justify exposure–response functions used.	Describe exposure-response functions used and health response studies used for modeling
Describe health response studies used for modeling (e.g., description of sample size, location, timeframe) and justify if health responses were not obtained from systematic reviews or meta-analyses (e.g., use of local studies).	
Define and justify metric(s) for measuring the health of populations (e.g., health metrics such as DALYs, years of life lost (YLL), years lived with disability (YLD), mortality, LLE, hospitalizations, emergency department visits, among others) appropriate for the specified causal pathways and target audience/decision makers.	Define and justify metric(s) for measuring the health of populations (e.g., health metrics such as DALYs, years of life lost (YLL), years lived with disability (YLD), mortality, LLE, hospitalizations, emergency department visits, among others) appropriate for the specified causal pathways and target audience/decision makers.
Conduct a sensitivity analysis and describe methods used.	Conduct a sensitivity analysis and describe methods used.
Describe general study limitations qualitatively (e.g., describe potential sources of bias and likely impact on findings) and quantitatively (e.g., describe limitations in valid parameterization of model).	Discuss general study limitations and uncertainty qualitatively (e.g., describe potential sources of bias and likely impact on findings) and quantitatively (e.g., describe limitations in valid parameterization of model).
Discuss sources of uncertainty that could not be addressed quantitatively.	
Discuss adverse consequences of mitigation actions (potential, hypothetical, or those observed in results) either qualitatively or quantitatively.	Discuss adverse consequences of mitigation actions (potential, hypothetical, or those observed in results) either qualitatively or quantitatively.
When legally and ethically possible, openly and publicly share data and code used for HEM studies to facilitate model validation of models by external researchers, collaboration between investigators, and the production of meta-analyses (including through retrospective harmonization), and new studies. If there are legal or ethical limitations to data sharing, these should be explicitly stated.	When legally and ethically possible, openly and publicly share data and code used for HEM studies to facilitate model validation of models by external researchers, collaboration between investigators, and the production of meta-analyses (including through retrospective harmonization), and new studies. If there are legal or ethical limitations to data sharing, these should be explicitly stated.

Table S5. Full description of included studies

CCS: Carbon capture and storage

Author, date	Region & Time scale	Modelling :<br>1. Exposure<br>2. Health<br>3. Economic	Sector of emission	- Health outcome<br>- Age range	Category of net-zero scenario(s)	Confidence assessment score
Air pollution						
(Cai et al., 2018)	China, 2010-2050	1. WRF/ CMAQ<br>2. Lifetables (IHIA)<br>3. VSL	Energy	- Mortality<br>- All	Not detailed : NDCs	Yes: 8<br>Yes partially: 2<br>Unclear: 0<br>No: 3
(Chen et al., 2020)	China, 2015-2050	1. LEAP<br>2. CRA (IF)<br>3. CCE	All-encompassing	- Mortality/ Morbidity<br>- All	Energy decarbonisation (renewable & CCS)	Yes: 7<br>Yes partially: 1<br>Unclear: 0<br>No: 5
(Chen et al., 2018)	China, 2015-2055	1. CMIP5<br>2. CRA	All-encompassing	- Mortality<br>- All	Not detailed : RCP	Yes: 1<br>Yes partially: 9<br>Unclear: 0<br>No: 3
(Cheng et al., 2023)	China, 2020-2060	1. WRF/ CMAQ / GCAM / DPEC<br>2. CRA	Energy, industry, transport, housing	- Mortality<br>- All	Energy decarbonisation (CCS)	Yes: 10<br>Yes partially: 1<br>Unclear: 0<br>No: 2
(Conibear et al., 2022)	China, 2020-2050	1. WRF/ ECLIPSE/ GAINS<br>2. CRA (PAF/GEMM)	Residential, industry, transport, agriculture, energy	- Mortality<br>- 25+	Not detailed: SSP-RCP	Yes: 7<br>Yes partially: 2<br>Unclear: 0<br>No: 4
(Dimitrova et al., 2021)	India, 2010-2050	1. GAINS<br>2. CRA (GEMM)	Energy, transport, industry, agriculture	- Mortality/ Life expectancy<br>- All	Not detailed: NDC & Target temperature	Yes: 9<br>Yes partially: 1<br>Unclear: 0<br>No: 3
(Hata et al., 2023)	Japan, 2015-2050	1. AIM/ GAINS/ CMAQ<br>2. Lifetables (BenMap)	All-encompassing	- Mortality<br>- All	Energy decarbonisation (Electrification & hydrogen)<br>Sufficiency	Yes: 5<br>Yes partially: 2<br>Unclear: 1<br>No: 5
(Li et al., 2019)	China, 2015-2050	1. TIMES/ GAINS<br>2. CRA	Energy, industry, transport, housing	- Mortality<br>- All	Not detailed: NDC & Target temperature	Yes: 7<br>Yes partially: 2<br>Unclear: 0<br>No: 4
(Lin et al., 2023)	Shaanxi (China), 2017-2060	1. CGE/ GAINS<br>2. CRA<br>3. VSL + COI	Service, transport, construction, industry, energy	- Mortality/ Morbidity<br>- All	Energy decarbonisation (carbon intensity & CCS)	Yes: 6<br>Yes partially: 1<br>Unclear: 0<br>No: 6
(Liu et al., 2022)	China, 2015-2050	1. GCAM/ DPEC/ WRF/ CMAQ<br>2. CRA	Energy, industry, housing, transport, food system, solvent	- Mortality<br>- 25+	Energy decarbonisation (CCS)	Yes: 11<br>Yes partially:<br>Unclear: 0<br>No: 2
(Lu et al., 2022)	Beijing (China), 2020-2050	1. GAINS<br>2. CRA (GEMM)<br>3. VSL + Social cost of carbon	Transport	- Mortality<br>- 25+	Energy decarbonisation (Electrification)/<br>Health in climate policies	Yes: 10<br>Yes partially: 1<br>Unclear: 0<br>No: 2
(Luo et al., 2023)	China, 2022-2050	1. GridPath<br>2. CRA (InMAP-China)<br>3. VSL	Energy	- None<br>- All	Energy decarbonisation (Renewable, CCS, nuclear)	Yes: 5<br>Yes partially: 3<br>Unclear: 1<br>No: 4
(X. Ma et al., 2023)	Shandong (China), 2017-2060	1. GCAM/ DPEC/ MEIC<br>2. CRA	Energy, industry, housing, transport, food system, solvent	- Acute myocardial infarction<br>- 18+	Energy decarbonisation (CCS)	Yes: 10<br>Yes partially: 0<br>Unclear: 1<br>No: 2
(T. Ma et al., 2023)	China, 2014-2060	1. IMED|TEC/ GAINS<br>2. CRA (GEMM & IMED|HEL)	Energy	- Mortality<br>- All	Energy decarbonisation (Electrification)	Yes: 8<br>Yes partially: 2<br>Unclear: 0<br>No: 3

(Markandya et al., 2018)	World, 2020-2050	1. GCAM<br>2. CRA (TM5/ FASST)<br>3. VSL	All-encompassing	- Mortality<br>- All	Not detailed (Target temperature)	Yes: 10<br>Yes partially: 1<br>Unclear: 0<br>No: 3
(McCollum et al., 2013)	World, 2010-2030	1. MESSAGE/ GAINS/ TMS<br>2. CRA (TM5)	Energy	- DALYs<br>- 30+	Health in climate policies	Yes: 6<br>Yes partially: 2<br>Unclear: 0<br>No: 5
(Mo et al., 2022)	Guangdong (China), 2015-2050	1. WRF/ MOSAIC<br>2. CRA	Energy	- Mortality<br>- 25+	Not detailed	Yes: 7<br>Yes partially: 2<br>Unclear: 0<br>No: 4
(Nawaz et al., 2023a)	41 countries, 2010-2040	1. GEOSChem<br>2. CRA	Agriculture, energy, industry, housing, shipping, transport, waste	- Mortality<br>- All	Not detailed	Yes: 8<br>Yes partially: 2<br>Unclear: 1<br>No: 2
(Nawaz et al., 2023b)	Santiago (Chile), 2016-2050	1. GEOSChem<br>2. CRA	Energy, industry, transport, agriculture, housing	- Mortality<br>- All	Health in climate policies	Yes: 8<br>Yes partially: 2<br>Unclear: 0<br>No: 3
(Ortiz et al., 2023)	Virginia (USA), 2016-2045	1. "Virginia Clean Economy Act<br>2. CRA	Energy	- Mortality<br>- 20+	Energy decarbonisation (Renewable)	Yes: 9<br>Yes partially: 1<br>Unclear: 0<br>No: 3
(Phillips, 2021)	South Korea, 2022-2050	1. National projections<br>2. Lifetables (AirQ+)	Energy, industry, transport, agriculture, waste, building	- Life-years<br>- 30+	Not detailed: NDC	Yes: 7<br>Yes partially: 3<br>Unclear: 0<br>No: 3
(Polonik et al., 2021)	World, 2018-2030	1. CMIP6 GISS-E2.1<br>2. CRA	Agriculture, energy, industry, transportation, residential/ commercial, solvents, waste, and shipping	- Mortality<br>- All	Health in climate policies	Yes: 8<br>Yes partially: 2<br>Unclear: 0<br>No: 3
(Qu et al., 2020)	China, 2015-2030	1. CREM/ CMAQ<br>2. CRA (CREM-HE)<br>3. VSL	Agriculture, energy, industry, transport, services	- Mortality/ Morbidity<br>- All	Not detailed: NDC & Target temperature	Yes: 6<br>Yes partially: 2<br>Unclear: 0<br>No: 5
(Rafaj et al., 2013)	EU-27, China, India, 2005-2050	1. POLES/ GAINS<br>2. Lifetables (GAINS)	Energy, industry, transport, housing	- Mortality/ Life expectancy<br>- All	Not detailed: Global CO2 reduction	Yes: 6<br>Yes partially: 1<br>Unclear: 0<br>No: 6
(Rafaj et al., 2021)	16 Asian country, 2015-2050	1. GAINS<br>2. CRA	Energy, industry, transport	- Mortality<br>- All	Not detailed: NDC & Target temperature	Yes: 9<br>Yes partially: 2<br>Unclear: 0<br>No: 2
(Rauner et al., 2020)	World, 2015-2050	1. REMIND/ GAINS<br>2. CRA (TM5-FASST)<br>3. VSL	All-encompassing	- Mortality<br>- All	Not detailed: NDC & Target temperature	Yes: 8<br>Yes partially: 2<br>Unclear: 0<br>No: 3
(Reddington et al., 2023)	World, 2020-2100	1. CMIP6<br>2. CRA (GEMM)	All-encompassing	- Mortality<br>- 25+	Not detailed	Yes: 8<br>Yes partially: 2<br>Unclear: 0<br>No: 3
(Reis et al., 2022)	World, 2015-2050	1. WITCH/ GAINS<br>2. CRA (FASST)	All-encompassing	- Mortality<br>- All	Financial instrument	Yes: 8<br>Yes partially: 2<br>Unclear: 0<br>No: 3
(Sampedro et al., 2020)	World, 2020-2050	1. GAINS/ TM5/ FASST<br>2. CRA (FASST)<br>3. VSL	Energy	- Mortality<br>- 30+ (IHD, stroke, COPD, LC)<br>0-5 (ALRI)	Energy decarbonisation (CCS & Nuclear)<br>Sufficiency	Yes: 10<br>Yes partially: 1<br>Unclear: 0<br>No: 2
(Sampedro et al., 2021)	World, 2020-2050	1. GAINS/ TM5/ FASST<br>2. CRA (FASST)	Energy, industry, housing, transport, shipping, land use, waste	- Mortality<br>- 30+ (IHD, stroke, COPD, LC)<br>0-5 (ALRI)	Health in climate policies	Yes: 9<br>Yes partially: 2<br>Unclear: 0<br>No: 2

(Schmid et al., 2019)	EU-28, 2015-2050	1. TIME PanEU<br>2. CRA (EcoSense)<br>3. VSL (EcoSense)	Energy	- None<br>- All	Energy decarbonisation (renewable)<br>Health in climate policies	Yes: 5<br>Yes partially: 2<br>Unclear: 1<br>No: 5
(Shen et al., 2022)	China, 2020-2060	1. MESSAGE/ GLOBIOM/ GAINS<br>2. CRA<br>3. VSL	Industry, transport, housing	- Mortality<br>- All	Energy decarbonisation	Yes: 6<br>Yes partially: 2<br>Unclear: 0<br>No: 5
(Shindell et al., 2018)	World, 2020-2100	1. GISS<br>2. CRA	All-encompassing	- Mortality<br>- All	Not detailed: Target temperature	Yes: 8<br>Yes partially: 2<br>Unclear: 0<br>No: 3
(Shindell et al., 2021)	China, 2020-2060	1. GISS<br>2. CRA<br>3. VSL	All-encompassing	- Mortality<br>- 30+	Not detailed: SSP-RCP	Yes: 7<br>Yes partially: 1<br>Unclear: 0<br>No: 5
(Tang et al., 2022)	China, 2010-2050	1. WRFChem/ GAINS<br>2. CRA (GEMM)<br>3. VSL	All-encompassing	- Mortality<br>- 25+	Not detailed: SSP-RCP	Yes: 10<br>Yes partially: 1<br>Unclear: 0<br>No: 2
(Vandyck et al., 2018)	World, 2020-2050	1. POLES-JRC/ ECLIPSE/ GAINS/ TM5/ FASST<br>2. CRA	All-encompassing	- Mortality<br>- All	Energy decarbonisation	Yes: 6<br>Yes partially: 2<br>Unclear: 0<br>No: 5
(Wang et al., 2020)	California (USA), 2010-2050	1. WRF-Chem/ MEET-CA<br>2. Lifetables (BenMap)<br>3. VSL	Energy	- Mortality<br>- All	Energy decarbonisation (renewable)	Yes: 10<br>Yes partially: 2<br>Unclear: 0<br>No: 1
(Wang et al., 2022)	World, 2015-2050	1. GAINS/ CAM-Chem<br>2. CRA (IMED|HEL)<br>3. VSL	All-encompassing	- Mortality<br>- All	Not detailed: NDC & Target temperature	Yes: 8<br>Yes partially: 2<br>Unclear: 0<br>No: 3
(H. Wang et al., 2023)	World, 2100-2020	1. GTIME/ GAINS<br>2. CRA (TM5/ FASST)<br>3. VSL	All-encompassing	- Mortality<br>- 30+ (IHD, cerebro-vascular, COPD, LC)<br>5+ (ALRI)	Not detailed: Target temperature	Yes: 6<br>Yes partially: 2<br>Unclear: 1<br>No: 4
(Y. Wang et al., 2023)	China, 2015-2060	1. GEOSChem<br>2. CRA (GEMM)	Agriculture, energy, industry, transport, residential, commercial, solvent, waste, shipping, natural burning	- Mortality<br>- 25+	Not detailed	Yes: 7<br>Yes partially: 2<br>Unclear: 0<br>No: 4
(Williams et al., 2018)	UK, 2011-2154	1. UK-TIMES/ ECLIPSE/ WRF/ CMAQ<br>2. Lifetables	Energy	- Life-years<br>- All	Energy decarbonisation (Nuclear)	Yes: 8<br>Yes partially: 1<br>Unclear: 0<br>No: 4
(Xie et al., 2021)	Anhui (China), 2020-2060	1. Emission factor<br>2. CRA<br>3. Unit value of health outcomes	Energy	- Mortality/ Morbidity<br>- All	Energy decarbonisation (Electrification)<br>Sufficiency	Yes: 8<br>Yes partially: 0<br>Unclear: 0<br>No: 5
(Xing et al., 2020)	China, 2015-2035	1. GCAM/ CMAQ<br>2. CRA<br>3. VSL (BenMap-CE)	Housing, industry, transport, energy	- Mortality<br>- All	Health in climate policies	Yes: 6<br>Yes partially: 3<br>Unclear: 0<br>No: 4
(Yang et al., 2019)	China, 2010-2050	1. China-MAPLE/ inF<br>2. CRA<br>3. VSL	Energy	- Mortality<br>- All	Energy decarbonisation (CCS)<br>Sufficiency	Yes: 7<br>Yes partially: 2<br>Unclear: 0<br>No: 4
(Silu Zhang et al., 2021)	Sichuan (China), 2015-2035	1. IMEG|CGE/ GAINS<br>2. CRA (IMED|HEL/ GEMM)<br>3. VSL + COI	Energy	- Mortality<br>- All	Energy decarbonisation (CCS)	Yes: 7<br>Yes partially: 1<br>Unclear: 0<br>No: 5

(Shihui Zhang et al., 2021)	China, 2020-2060	1. CHEER-LCT/AIR<br>2. CRA (CHEER-HA)<br>3. VSL + COI	Energy	- Mortality/ Life expectancy<br>- All	Energy decarbonisation (CCS & renewable)	Yes: 8<br>Yes partially: 0<br>Unclear: 0<br>No: 5
(Zhao et al., 2019)	California (USA), 2010-2050	1. WRF-Chem/ CMAQ<br>2. Lifetables (BenMap-CE)	Energy, transport, residential, commercial, industry, agriculture	- Mortality<br>- All	Health in climate policies<br>Financial instrument	Yes: 5<br>Yes partially: 1<br>Unclear: 0<br>No: 7
(Zhu et al., 2022)	California (USA), 2018-2050	1. WRF-Chem/ SMOKE/ CMAQ<br>2. Lifetables (BenMap-CE)	Transport, housing	- Mortality<br>- All	Energy decarbonisation (electrification)	Yes: 9<br>Yes partially: 0<br>Unclear: 0<br>No: 4
(Zyśk et al., 2020)	Poland, 2015-2050	1. Polyphemus<br>2. Life tables (πESA)<br>3. Unit damage costs	Energy	- Life-years / Morbidity<br>- All	Energy decarbonisation (renewable)	Yes: 6<br>Yes partially: 1<br>Unclear: 0<br>No: 6
(Zyśk et al., 2021)	Poland, 2018-2050	1. Polyphemus<br>2. Life tables<br>3. Unit damage costs	Energy	- None<br>- All	Energy decarbonisation (nuclear & renewable)	Yes: 6<br>Yes partially: 0<br>Unclear: 0<br>No: 7
**Air and indoor pollution**						
(Dimitrova et al., 2022)	India, 2010-2050	1. GAINS/ MESSAGE-GLOBIOM<br>2. Microsimulation	All-encompassing	- Child stunting<br>- 0-5	Health in climate policies	Yes: 11<br>Yes partially: 1<br>Unclear: 1<br>No: 0
(Jacobson et al., 2017)	139 countries	1. GATOR-GCMOM<br>2. CRA<br>3. VSL + morbidity	Energy	- Mortality	Energy decarbonisation (renewable)	Yes: 3<br>Yes partially: 1<br>Unclear: 3<br>No: 6
(Rafaj et al., 2018)	China, India, EU, Indonesia, South Africa, 2015-2040	1. WEM_GAINS<br>2. CRA	Energy, industry, transport, housing, agriculture, waste	- Mortality<br>- All	Health in climate policies	Yes: 6<br>Yes partially: 2<br>Unclear: 0<br>No: 5
**Indoor pollution**						
(Shrubsole et al., 2015)	London, Milton Keynes (UK), 2010-2050	1. SCRIBE<br>2. Lifetables (IOMLIFET)	Housing	- Life-years<br>- 40+	Energy decarbonisation (electrification)	Yes: 7<br>Yes partially: 2<br>Unclear: 0<br>No: 4
**Physical activity**						
(Barban et al., 2022)	France, 2021-2050	1. Negawatt<br>2. Lifetables<br>3. VSL	Transport	- Mortality/ Life expectancy/ Life-years<br>- 20-84	Sufficiency	Yes: 12<br>Yes partially: 0<br>Unclear: 0<br>No: 1
(Creutzig et al., 2012)	Barcelona, Freiburg, Malmö, Sofia, 2010-2040	1. Theoretical policies<br>2. CRA (HEAT)	Transport	- Mortality<br>- All	Health in climate policies<br>Financial instrument	Yes: 7<br>Yes partially: 1<br>Unclear: 0<br>No: 5
**Pollution, physical activity and diet**						
(Hamilton et al., 2021)	Brazil, China, Germany, India, Indonesia, Nigeria, South Africa, UK, USA, 2015-2040	1. GAINS (pollution) / IMPACT (diet)/ Theoretical policies (physical activity)<br>2. CRA	Energy/ Food system/ Transport	- Mortality<br>- 0-85 (air pollution)	Health in climate policies	Yes: 8<br>Yes partially: 2<br>Unclear: 0<br>No: 3
(Milner et al., 2023)	England, Wales, 2021-2100	1. CRAFT/ SCRIBE (pollution)<br>ITHIM (physical activity)<br>Theoretical policies (Diet)<br>2. Lifetables	Energy/ Food system/ Transport/ Housing	- Life-years<br>- 25+ (air pollution)/ 15+ (physical activity)	Energy decarbonisation<br>Sufficiency	Yes: 9<br>Yes partially: 2<br>Unclear: 0<br>No: 2

Figure S1. Typology of net-zero and baseline scenarios.

Regular style is used for baseline scenarios that assume increasing GHG emissions. Underlined text is used when decreasing GHG emissions over time are assumed. Bold text is used for baseline scenarios that refer to a reference year as a baseline exposure.

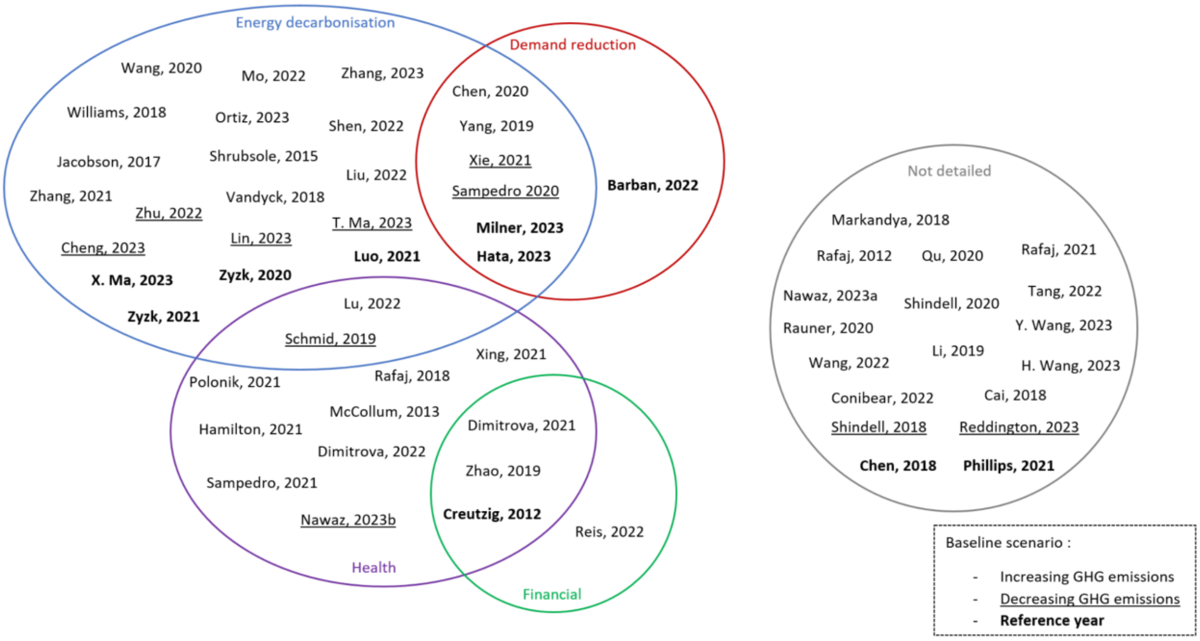

Figure S2. Preventable mortality fraction across net-zero scenarios for air pollution exposure, by health impact assessment methods (A) and choice in the baseline scenario (B)

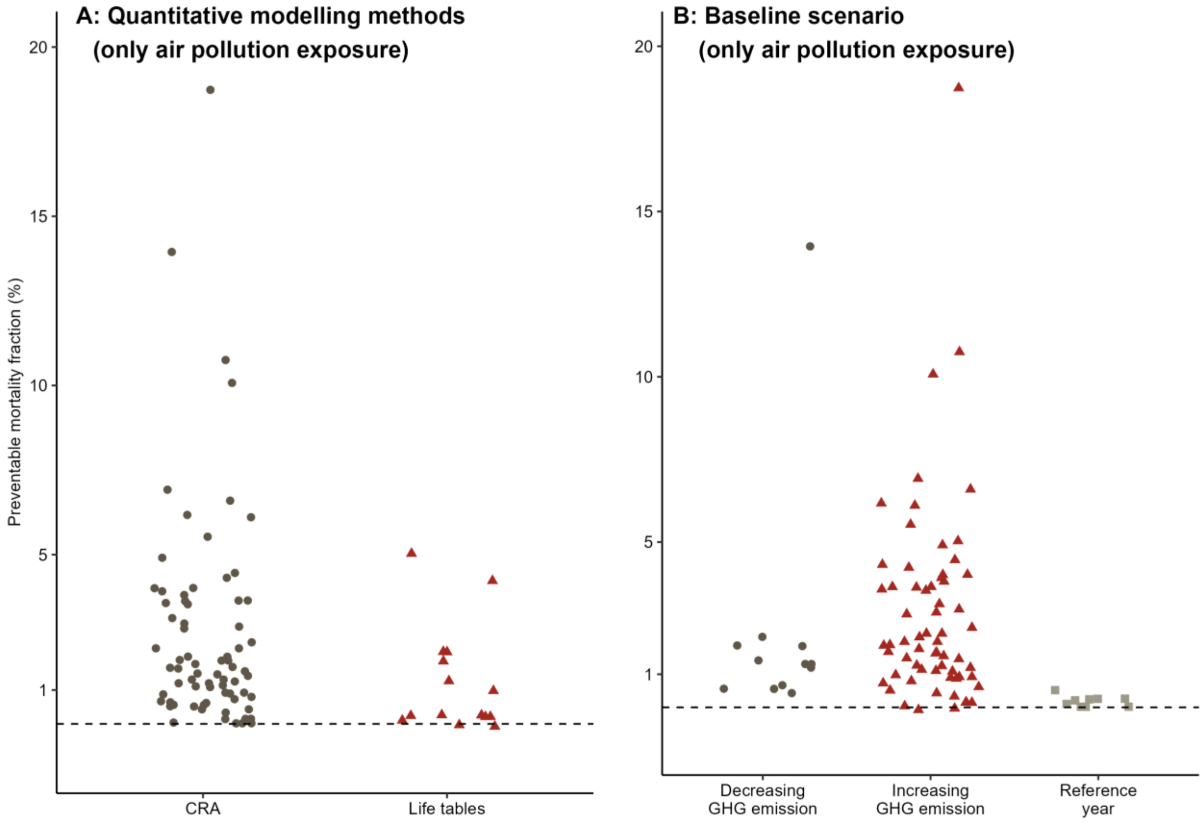

Figure S3. Health impact of investigations led in China

Horizontal bar represents the median value of preventable mortality (%)

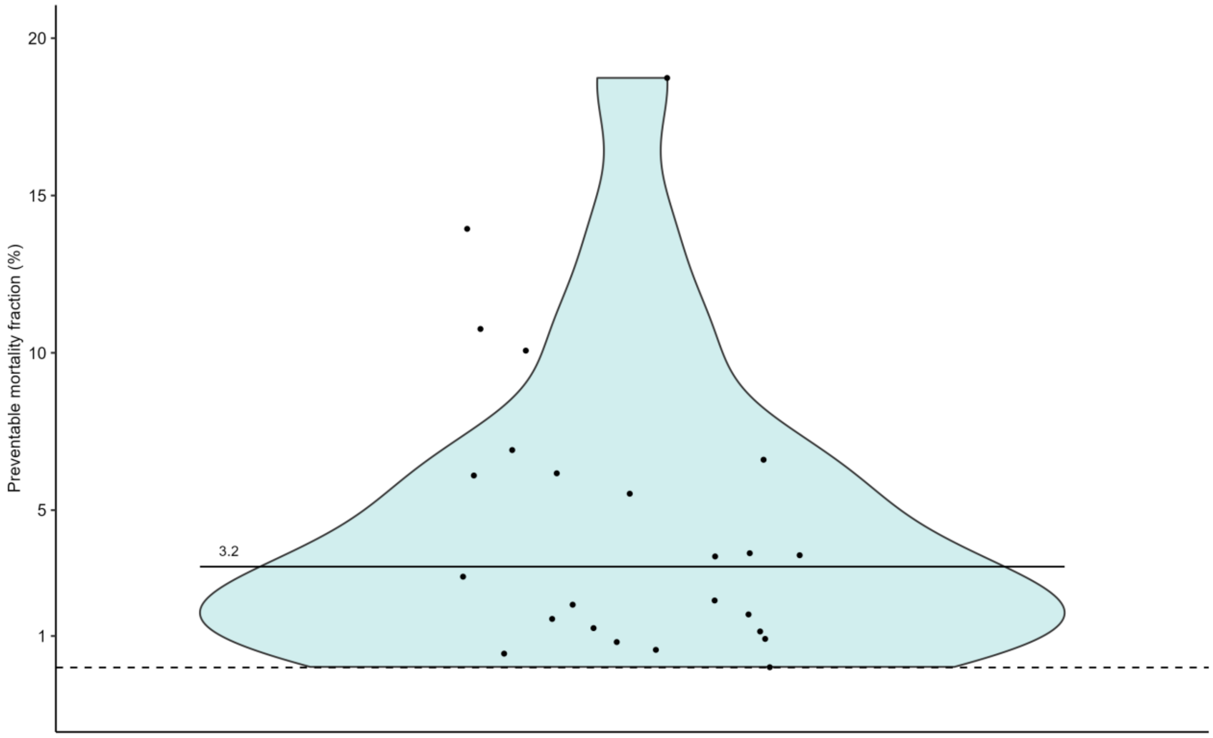

Figure S4. Life-years gained across net-zero scenarios for total sample

Horizontal bar represents the median value of life-years gained per 100 000

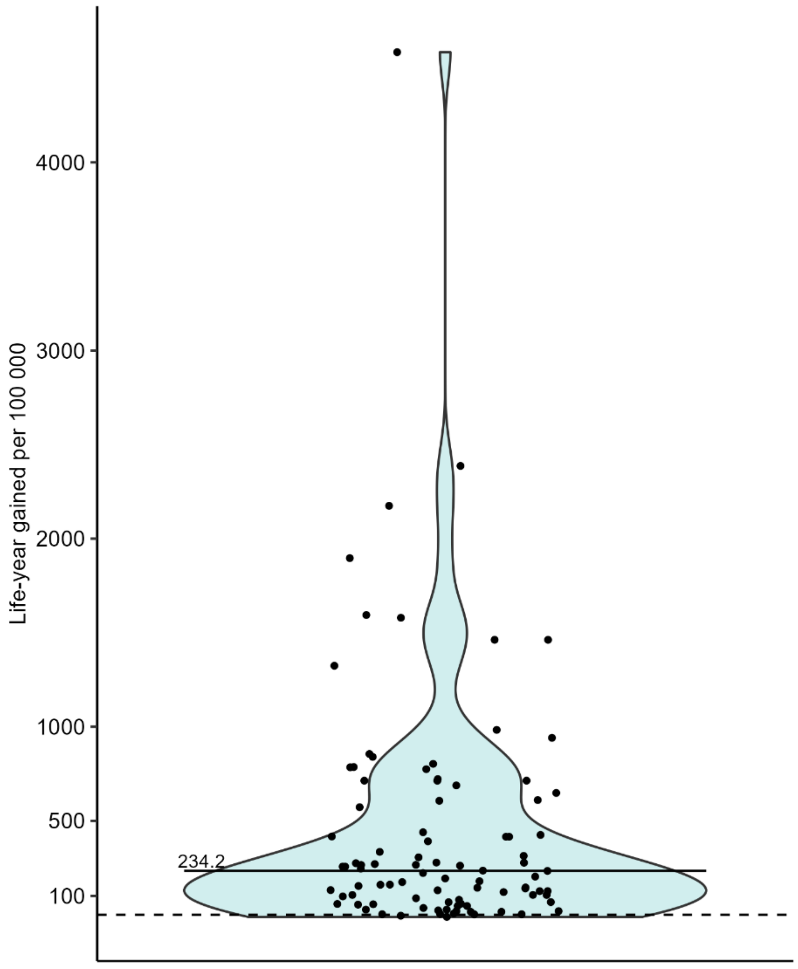

Figure S5. Countries of 1st authors' institution

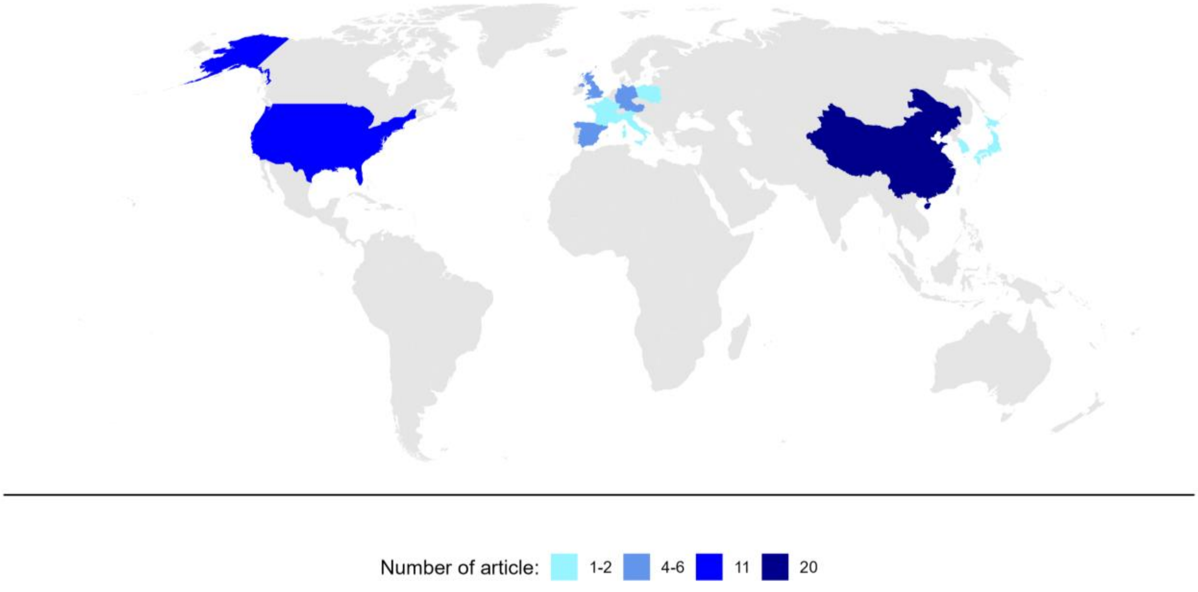

References

Barban, P., De Nazelle, A., Chatelin, S., Quirion, P., Jean, K., 2022. Assessing the Health Benefits of Physical Activity Due to Active Commuting in a French Energy Transition Scenario. Int. J. Public Health 67, 1605012. https://doi.org/10.3389/ijph.2022.1605012

Cai, W., Hui, J., Wang, C., Zheng, Y., Zhang, X., Zhang, Q., Gong, P., 2018. The Lancet Countdown on PM 2·5 pollution-related health impacts of China's projected carbon dioxide mitigation in the electric power generation sector under the Paris Agreement: a modelling study. Lancet Planet. Health 2, e151–e161. https://doi.org/10.1016/S2542-5196(18)30050-0

California Department of Finance. Demographic Research Unit., 2023. Report P-1A: Total Population Projections, California, 2020-2060 (Baseline 2019 Population Projections; Vintage 2023 Release). [WWW Document]. URL https://dof.ca.gov/forecasting/demographics/projections/

Chen, H., Wang, Z., Xu, S., Zhao, Y., Cheng, Q., Zhang, B., 2020. Energy demand, emission reduction and health co-benefits evaluated in transitional China in a 2 °C warming world. J. Clean. Prod. 264. https://doi.org/10.1016/j.jclepro.2020.121773

Chen, K., Fiore, A.M., Chen, R., Jiang, L., Jones, B., Schneider, A., Peters, A., Bi, J., Kan, H., Kinney, P.L., 2018. Future ozone-related acute excess mortality under climate and population change scenarios in China: A modeling study. PLOS Med. 15, e1002598. https://doi.org/10.1371/journal.pmed.1002598

Cheng, J., Tong, D., Liu, Y., Geng, G., Davis, S.J., He, K., Zhang, Q., 2023. A synergistic approach to air pollution control and carbon neutrality in China can avoid millions of premature deaths annually by 2060. One Earth 6, 978–989. https://doi.org/10.1016/j.oneear.2023.07.007

Conibear, L., Reddington, C.L., Silver, B.J., Arnold, S.R., Turnock, S.T., Klimont, Z., Spracklen, D.V., 2022. The contribution of emission sources to the future air pollution disease burden in China. Environ. Res. Lett. 17, 064027. https://doi.org/10.1088/1748-9326/ac6f6f

Creutzig, F., Mühlhoff, R., Römer, J., 2012. Decarbonizing urban transport in European cities: four cases show possibly high co-benefits. Environ. Res. Lett. 7, 044042. https://doi.org/10.1088/1748-9326/7/4/044042

Dimitrova, A., Marois, G., Kiesewetter, G., K C, S., Rafaj, P., Tonne, C., 2021. Health impacts of fine particles under climate change mitigation, air quality control, and demographic change in India. Environ. Res. Lett. 16. https://doi.org/10.1088/1748-9326/abe5d5

Dimitrova, A., Marois, G., Kiesewetter, G., Rafaj, P., Pachauri, S., KC, S., Olmos, S., Rasella, D., Tonne, C., 2022. Projecting the impact of air pollution on child stunting in India—synergies and trade-offs between climate change mitigation, ambient air quality control, and clean cooking access. Environ. Res. Lett. 17. https://doi.org/10.1088/1748-9326/ac8e89

Hamilton, I., Kennard, H., McGushin, A., Höglund-Isaksson, L., Kiesewetter, G., Lott, M., Milner, J., Purohit, P., Rafaj, P., Sharma, R., Springmann, M., Woodcock, J., Watts, N., 2021. The public health implications of the Paris Agreement: a modelling study. Lancet Planet. Health 5, e74–e83. https://doi.org/10.1016/S2542-5196(20)30249-7

Hata, H., Inoue, K., Yoshikado, H., Genchi, Y., Tsunemi, K., 2023. Impact of introducing net-zero carbon strategies on tropospheric ozone (O3) and fine particulate matter (PM2.5) concentrations in Japanese region in 2050. Sci. Total Environ. 891, 164442. https://doi.org/10.1016/j.scitotenv.2023.164442

Hess, J.J., Ranadive, N., Boyer, C., Aleksandrowicz, L., Anenberg, S.C., Aunan, K., Belesova, K., Bell, M.L., Bickersteth, S., Bowen, K., Burden, M., Campbell-Lendrum, D., Carlton, E., Cissé, G., Cohen, F., Dai, H., Dangour, A.D., Dasgupta, P., Frumkin, H., Gong, P., Gould, R.J., Haines, A., Hales, S., Hamilton, I., Hasegawa, T., Hashizume, M., Honda, Y., Horton, D.E., Karambelas, A., Kim, H., Kim, S.E., Kinney, P.L., Kone, I., Knowlton, K., Lelieveld, J., Limaye, V.S., Liu, Q., Madaniyazi, L., Martinez, M.E., Mauzerall, D.L., Milner, J., Neville, T., Nieuwenhuijsen, M., Pachauri, S., Perera, F., Pineo, H., Remais, J.V., Saari, R.K., Sampedro, J., Scheelbeek, P., Schwartz, J., Shindell, D., Shyamsundar, P., Taylor, T.J., Tonne, C., Van Vuuren, D., Wang, C., Watts, N., West, J.J., Wilkinson, P., Wood, S.A., Woodcock, J., Woodward, A., Xie, Y., Zhang,

Y., Ebi, K.L., 2020. Guidelines for Modeling and Reporting Health Effects of Climate Change Mitigation Actions. Environ. Health Perspect. 128, 115001. https://doi.org/10.1289/EHP6745
Institute for Health Metrics and Evaluation (IHME), 2024. Global Burden of Disease Collaborative Network (GBD 2021).
Jacobson, M.Z., Delucchi, M.A., Bauer, Z.A.F., Goodman, S.C., Chapman, W.E., Cameron, M.A., Bozonnat, C., Chobadi, L., Clonts, H.A., Enevoldsen, P., Erwin, J.R., Fobi, S.N., Goldstrom, O.K., Hennessy, E.M., Liu, J., Lo, J., Meyer, C.B., Morris, S.B., Moy, K.R., O'Neill, P.L., Petkov, I., Redfern, S., Schucker, R., Sontag, M.A., Wang, J., Weiner, E., Yachanin, A.S., 2017. 100% Clean and Renewable Wind, Water, and Sunlight All-Sector Energy Roadmaps for 139 Countries of the World. Joule 1, 108–121. https://doi.org/10.1016/j.joule.2017.07.005
Li, N., Chen, W., Rafaj, P., Kiesewetter, G., Schöpp, W., Wang, H., Zhang, H., Krey, V., Riahi, K., 2019. Air Quality Improvement Co-benefits of Low-Carbon Pathways toward Well Below the 2 °C Climate Target in China. Environ. Sci. Technol. 53, 5576–5584. https://doi.org/10.1021/acs.est.8b06948
Lin, Z., Wang, P., Ren, S., Zhao, D., 2023. Comprehensive impact assessment of carbon neutral pathways and air pollution control policies in Shaanxi Province of China. Resour. Conserv. Recycl. Adv. 18. https://doi.org/10.1016/j.rcradv.2023.200143
Liu, Y., Tong, D., Cheng, J., Davis, S.J., Yu, S., Yarlagadda, B., Clarke, L.E., Brauer, M., Cohen, A.J., Kan, H., Xue, T., Zhang, Q., 2022. Role of climate goals and clean-air policies on reducing future air pollution deaths in China: a modelling study. Lancet Planet. Health 6, e92–e99. https://doi.org/10.1016/S2542-5196(21)00326-0
Lu, C., Adger, W.N., Morrissey, K., Zhang, S., Venevsky, S., Yin, H., Sun, T., Song, X., Wu, C., Dou, X., Zhu, B., Liu, Z., 2022. Scenarios of demographic distributional aspects of health co-benefits from decarbonising urban transport. Lancet Planet. Health 6, e461–e474. https://doi.org/10.1016/S2542-5196(22)00089-4
Luo, Q., Garcia-Menendez, F., Lin, J., He, G., Johnson, J.X., 2023. Accelerating China's power sector decarbonization can save lives: integrating public health goals into power sector planning decisions. Environ. Res. Lett. 18, 104023. https://doi.org/10.1088/1748-9326/acf84b
Ma, T., Zhang, S., Xiao, Y., Liu, X., Wang, M., Wu, K., Shen, G., Huang, C., Fang, Y.R., Xie, Y., 2023. Costs and health benefits of the rural energy transition to carbon neutrality in China. Nat. Commun. 14, 6101. https://doi.org/10.1038/s41467-023-41707-7
Ma, X., Zhang, B., Duan, H., Wu, H., Dong, J., Guo, X., Lu, Z., Ma, J., Xi, B., 2023. Estimating future PM2.5-attributed acute myocardial infarction incident cases under climate mitigation and population change scenarios in Shandong Province, China. Ecotoxicol. Environ. Saf. 256, 114893. https://doi.org/10.1016/j.ecoenv.2023.114893
Markandya, A., Sampedro, J., Smith, S.J., Van Dingenen, R., Pizarro-Irizar, C., Arto, I., González-Eguino, M., 2018. Health co-benefits from air pollution and mitigation costs of the Paris Agreement: a modelling study. Lancet Planet. Health 2, e126–e133. https://doi.org/10.1016/S2542-5196(18)30029-9
McCollum, D.L., Krey, V., Riahi, K., Kolp, P., Grubler, A., Makowski, M., Nakicenovic, N., 2013. Climate policies can help resolve energy security and air pollution challenges. Clim. Change 119, 479–494. https://doi.org/10.1007/s10584-013-0710-y
Milner, J., Turner, G., Ibbetson, A., Eustachio Colombo, P., Green, R., Dangour, A.D., Haines, A., Wilkinson, P., 2023. Impact on mortality of pathways to net zero greenhouse gas emissions in England and Wales: a multisectoral modelling study. Lancet Planet. Health 7, e128–e136. https://doi.org/10.1016/S2542-5196(22)00310-2
Mo, H., Jiang, K., Wang, P., Shao, M., Wang, X., 2022. Co-Benefits of Energy Structure Transformation and Pollution Control for Air Quality and Public Health until 2050 in Guangdong, China. Int. J. Environ. Res. Public. Health 19, 14965. https://doi.org/10.3390/ijerph192214965
Nawaz, M.O., Henze, D.K., Anenberg, S.C., Braun, C., Miller, J., Pronk, E., 2023a. A Source Apportionment and Emission Scenario Assessment of PM 2.5 and O3 related Health Impacts in G20 Countries. GeoHealth 7, e2022GH000713. https://doi.org/10.1029/2022GH000713

Nawaz, M.O., Henze, D.K., Huneeus, N.J., Osses, M., Álamos, N., Opazo, M.A., Gallardo, L., 2023b. Sources of Air Pollution Health Impacts and Co-Benefits of Carbon Neutrality in Santiago, Chile. J. Geophys. Res. Atmospheres 128, e2023JD038808. https://doi.org/10.1029/2023JD038808

Ortiz, L.E., Stiles, R., Whitaker, S., Maibach, E., Kinter, J., Henneman, L., Krall, J., Bubbosh, P., Cash, B., 2023. Public health benefits of zero-emission electric power generation in Virginia. Heliyon 9, e20198. https://doi.org/10.1016/j.heliyon.2023.e20198

Page, M.J., McKenzie, J.E., Bossuyt, P.M., Boutron, I., Hoffmann, T.C., Mulrow, C.D., Shamseer, L., Tetzlaff, J.M., Akl, E.A., Brennan, S.E., Chou, R., Glanville, J., Grimshaw, J.M., Hróbjartsson, A., Lalu, M.M., Li, T., Loder, E.W., Mayo-Wilson, E., McDonald, S., McGuinness, L.A., Stewart, L.A., Thomas, J., Tricco, A.C., Welch, V.A., Whiting, P., Moher, D., 2021. The PRISMA 2020 statement: an updated guideline for reporting systematic reviews. BMJ n71. https://doi.org/10.1136/bmj.n71

Phillips, D., 2021. Ambient Air Quality Synergies with a 2050 Carbon Neutrality Pathway in South Korea. Climate 10, 1. https://doi.org/10.3390/cli10010001

Polonik, P., Ricke, K., Burney, J., 2021. Paris Agreement's Ambiguity About Aerosols Drives Uncertain Health and Climate Outcomes. Earths Future 9. https://doi.org/10.1029/2020EF001787

Qu, C., Yang, X., Zhang, D., Zhang, X., 2020. ESTIMATING HEALTH CO-BENEFITS OF CLIMATE POLICIES IN CHINA: AN APPLICATION OF THE REGIONAL EMISSIONS-AIR QUALITY-CLIMATE-HEALTH (REACH) FRAMEWORK. Clim. Change Econ. 11, 2041004. https://doi.org/10.1142/S2010007820410043

Rafaj, P., Kiesewetter, G., Gül, T., Schöpp, W., Cofala, J., Klimont, Z., Purohit, P., Heyes, C., Amann, M., Borken-Kleefeld, J., Cozzi, L., 2018. Outlook for clean air in the context of sustainable development goals. Glob. Environ. Change 53, 1–11. https://doi.org/10.1016/j.gloenvcha.2018.08.008

Rafaj, P., Kiesewetter, G., Krey, V., Schoepp, W., Bertram, C., Drouet, L., Fricko, O., Fujimori, S., Harmsen, M., Hilaire, J., Huppmann, D., Klimont, Z., Kolp, P., Aleluia Reis, L., Van Vuuren, D., 2021. Air quality and health implications of 1.5 °c-2 °c climate pathways under considerations of ageing population: A multi-model scenario analysis. Environ. Res. Lett. 16. https://doi.org/10.1088/1748-9326/abdf0b

Rafaj, P., Schöpp, W., Russ, P., Heyes, C., Amann, M., 2013. Co-benefits of post-2012 global climate mitigation policies. Mitig. Adapt. Strateg. Glob. Change 18, 801–824. https://doi.org/10.1007/s11027-012-9390-6

Rauner, S., Hilaire, J., Klein, D., Strefler, J., Luderer, G., 2020. Air quality co-benefits of ratcheting up the NDCs. Clim. Change 163, 1481–1500. https://doi.org/10.1007/s10584-020-02699-1

Reddington, C.L., Turnock, S.T., Conibear, L., Forster, P.M., Lowe, J.A., Ford, L.B., Weaver, C., Van Bavel, B., Dong, H., Alizadeh, M.R., Arnold, S.R., 2023. Inequalities in Air Pollution Exposure and Attributable Mortality in a Low Carbon Future. Earths Future 11, e2023EF003697. https://doi.org/10.1029/2023EF003697

Reis, L.A., Drouet, L., Tavoni, M., 2022. Internalising health-economic impacts of air pollution into climate policy: a global modelling study. Lancet Planet. Health 6, e40–e48. https://doi.org/10.1016/S2542-5196(21)00259-X

Sampedro, J., Cui, R.Y., McJeon, H., Smith, S.J., Hultman, N., He, L., Sen, A., Van Dingenen, R., Cazcarro, I., 2021. Quantifying the reductions in mortality from air-pollution by cancelling new coal power plants. Energy Clim. Change 2. https://doi.org/10.1016/j.egycc.2020.100023

Sampedro, J., Smith, S.J., Arto, I., González-Eguino, M., Markandya, A., Mulvaney, K.M., Pizarro-Irizar, C., Van Dingenen, R., 2020. Health co-benefits and mitigation costs as per the Paris Agreement under different technological pathways for energy supply. Environ. Int. 136, 105513. https://doi.org/10.1016/j.envint.2020.105513

Schmid, D., Korkmaz, P., Blesl, M., Fahl, U., Friedrich, R., 2019. Analyzing transformation pathways to a sustainable European energy system—Internalization of health damage costs caused by air pollution. Energy Strategy Rev. 26. https://doi.org/10.1016/j.esr.2019.100417

Shen, J., Cai, W., Chen, X., Chen, X., Zhao, Z., Ma, Z., Yang, F., Zhang, S., 2022. Synergies of carbon neutrality, air pollution control, and health improvement — a case study of China energy interconnection scenario. Glob. Energy Interconnect. 5, 531–542. https://doi.org/10.1016/j.gloei.2022.10.007

Shindell, D., Faluvegi, G., Seltzer, K., Shindell, C., 2018. Quantified, localized health benefits of accelerated carbon dioxide emissions reductions. Nat. Clim. Change 8, 291–295. https://doi.org/10.1038/s41558-018-0108-y

Shindell, D., Ru, M., Zhang, Y., Seltzer, K., Faluvegi, G., Nazarenko, L., Schmidt, G.A., Parsons, L., Challapalli, A., Yang, L., Glick, A., 2021. Temporal and spatial distribution of health, labor, and crop benefits of climate change mitigation in the United States. Proc. Natl. Acad. Sci. U. S. A. 118. https://doi.org/10.1073/pnas.2104061118

Shrubsole, C., Das, P., Milner, J., Hamilton, I.G., Spadaro, J.V., Oikonomou, E., Davies, M., Wilkinson, P., 2015. A tale of two cities: Comparison of impacts on CO2 emissions, the indoor environment and health of home energy efficiency strategies in London and Milton Keynes. Atmos. Environ. 120, 100–108. https://doi.org/10.1016/j.atmosenv.2015.08.074

Tang, R., Zhao, J., Liu, Y., Huang, X., Zhang, Y., Zhou, D., Ding, A., Nielsen, C.P., Wang, H., 2022. Air quality and health co-benefits of China's carbon dioxide emissions peaking before 2030. Nat. Commun. 13, 1008. https://doi.org/10.1038/s41467-022-28672-3

Vandyck, T., Keramidas, K., Kitous, A., Spadaro, J.V., Van Dingenen, R., Holland, M., Saveyn, B., 2018. Air quality co-benefits for human health and agriculture counterbalance costs to meet Paris Agreement pledges. Nat. Commun. 9, 4939. https://doi.org/10.1038/s41467-018-06885-9

Wang, H., Chen, W., Rauner, S., Bertram, C., Luderer, G., Kriegler, E., 2023. The Double Dividend of International Cooperation for Climate Mitigation Cost Effectiveness and Public Health Cobenefits. Environ. Sci. Technol. 57, 4061–4070. https://doi.org/10.1021/acs.est.2c08326

Wang, T., Jiang, Z., Zhao, B., Gu, Y., Liou, K.-N., Kalandiyur, N., Zhang, D., Zhu, Y., 2020. Health co-benefits of achieving sustainable net-zero greenhouse gas emissions in California. Nat. Sustain. 3, 597–605. https://doi.org/10.1038/s41893-020-0520-y

Wang, Y., Liao, H., Chen, H., Chen, L., 2023. Future Projection of Mortality From Exposure to PM $_{2.5}$ and O $_3$ Under the Carbon Neutral Pathway: Roles of Changing Emissions and Population Aging. Geophys. Res. Lett. 50, e2023GL104838. https://doi.org/10.1029/2023GL104838

Wang, Y., Xie, M., Wu, Y., Zhang, X., Wang, M., Zhang, Y., Xie, Y., 2022. Ozone-related Co-benefits of China's Climate mitigation Policy. Resour. Conserv. Recycl. 182. https://doi.org/10.1016/j.resconrec.2022.106288

Williams, M.L., Lott, M.C., Kitwiroon, N., Dajnak, D., Walton, H., Holland, M., Pye, S., Fecht, D., Toledano, M.B., Beevers, S.D., 2018. The Lancet Countdown on health benefits from the UK Climate Change Act: a modelling study for Great Britain. Lancet Planet. Health 2, e202–e213. https://doi.org/10.1016/S2542-5196(18)30067-6

Xie, W., Guo, W., Shao, W., Li, F., Tang, Z., 2021. Environmental and health co-benefits of coal regulation under the carbon neutral target: A case study in Anhui province, China. Sustain. Switz. 13. https://doi.org/10.3390/su13116498

Xing, J., Lu, X., Wang, S., Wang, T., Ding, D., Yu, S., Shindell, D., Ou, Y., Morawska, L., Li, S., Ren, L., Zhang, Y., Loughlin, D., Zheng, H., Zhao, B., Liu, S., Smith, K.R., Hao, J., 2020. The quest for improved air quality may push China to continue its CO2 reduction beyond the Paris Commitment. Proc. Natl. Acad. Sci. 117, 29535–29542. https://doi.org/10.1073/pnas.2013297117

Yang, X., Xi, X., Lin, W., Guo, S., 2019. Effect of China's energy conservation efforts on reducing health damage. Energy Procedia 158, 3768–3773. https://doi.org/10.1016/j.egypro.2019.01.878

Zhang, Silu, Wu, Y., Liu, X., Qian, J., Chen, J., Han, L., Dai, H., 2021. Co-benefits of deep carbon reduction on air quality and health improvement in Sichuan Province of China. Environ. Res. Lett. 16, 095011. https://doi.org/10.1088/1748-9326/ac1133

Zhang, Shihui, An, K., Li, J., Weng, Y., Zhang, Shaohui, Wang, S., Cai, W., Wang, C., Gong, P., 2021. Incorporating health co-benefits into technology pathways to achieve China's 2060 carbon

neutrality goal: a modelling study. Lancet Planet. Health 5, e808–e817. https://doi.org/10.1016/S2542-5196(21)00252-7
Zhao, B., Wang, T., Jiang, Z., Gu, Y., Liou, K.-N., Kalandiyur, N., Gao, Y., Zhu, Y., 2019. Air Quality and Health Cobenefits of Different Deep Decarbonization Pathways in California. Environ. Sci. Technol. 53, 7163–7171. https://doi.org/10.1021/acs.est.9b02385
Zhu, S., Mac Kinnon, M., Carlos-Carlos, A., Davis, S.J., Samuelsen, S., 2022. Decarbonization will lead to more equitable air quality in California. Nat. Commun. 13, 5738. https://doi.org/10.1038/s41467-022-33295-9
Zyśk, J., Wyrwa, A., Pluta, M., Olkuski, T., Suwała, W., Raczyński, M., 2021. The health impact and external cost of electricity production. Energies 14. https://doi.org/10.3390/en14248263
Zyśk, J., Wyrwa, A., Suwała, W., Pluta, M., Olkuski, T., Raczyński, M., 2020. The impact of decarbonization scenarios on air quality and human health in Poland-analysis of scenarios up to 2050. Atmosphere 11. https://doi.org/10.3390/atmos11111222
