# Supplementary material for: The public health co-benefits of strategies consistent with net-zero emissions: a systematic review
Source: Lancet Planet Health. Author manuscript; Available in PMC 2025 Dec 1. (PMC7618420; doi:10.1016/S2542-5196(24)00330-9)
Supplement: Supplementary Materials [file EMS211168-supplement-Supplementary_Materials.zip › 1-s2.0-S2542519624003309-mmc1.pdf]

THE LANCET
Planetary Health

Supplementary appendix 1

This translation in French was submitted by the authors and we reproduce it as supplied. It has not been peer reviewed. *The Lancet*'s editorial processes have only been applied to the original in English, which should serve as reference for this manuscript.

Cette traduction en français a été proposée par les auteurs et nous l'avons reproduite telle quelle. Elle n'a pas été examinée par des pairs. Les processus éditoriaux du *Lancet* n'ont été appliqués qu'à l'original en anglais et c'est cette version qui doit servir de référence pour ce manuscrit.

Supplement to: Moutet L, Bernard P, Green R, et al. The public health co-benefits of strategies consistent with net-zero emissions: a systematic review. *Lancet Planet Health* 2025; published online Feb 12. https://doi.org/10.1016/S2542-5196(24)00330-9.

Les co-bénéfices sanitaires des stratégies compatibles avec la neutralité carbone: une revue systématique d'études quantitatives

Léo Moutet*, Paquito Bernard, Rosemary Green, James Milner, Andy Haines, Rémy Slama, Laura Temime, et Kévin Jean

* Auteur correspondent. Email: leo.moutet@gmail.com

RESUME

La transition des sociétés vers la neutralité carbone (zéro émission nette) devrait générer des co-bénéfices sanitaires significatifs. Toutefois, l'ampleur de ces bénéfices reste peu documentée et peut varier selon le contexte. Une synthèse des preuves scientifiques disponibles sur ces co-bénéfices pourrait renforcer l'engagement des décideurs et des populations dans les actions d'atténuation du changement climatique. Nous avons mené une revue systématique incluant 58 études quantitatives explorant 125 scénarios de neutralité en carbone. Par l'amélioration de la qualité de l'air, de l'activité physique et des changements alimentaires, des co-bénéfices sanitaires important ont été identifiés. La moitié des scénarios analysés montrent une réduction de la mortalité supérieure à 1,5 %, soit un gain de 234 années de vie pour 100 000 habitants, en plus des bénéfices directement liés à la stabilisation du climat. Parmi 96 scénarios étudiés, 94 indiquent que les politiques atteignant la neutralité carbone entraîneraient des bénéfices sanitaires majeurs. Pour 34 des 35 études nationales ou régionales, les politiques de neutralité carbone apporteraient des bénéfices significatifs sur la qualité de l'air local, indépendamment des actions mises en œuvre dans les pays voisins. Cependant, ces co-bénéfices varient selon les secteurs d'émissions explorés, les leviers de décarbonation, les approches de modélisation et les localisations. Parmi les études comprenant une analyse coût-bénéfice, 11 sur 13 estiment que les bénéfices monétisés excèdent les coûts d'implémentation des politiques climatiques. Cette revue met en évidence la nécessité de développer un cadre standardisé pour évaluer et comparer les impacts sanitaires des actions d'atténuation à travers les secteurs d'émission. Elle confirme également que l'atteinte des objectifs de neutralité carbone soutient des politiques de santé publique ambitieuses, notamment dans les pays à fortes émissions.

Mots-clés: Évaluation d'impacts sanitaires, trajectoires vers la neutralité carbone, revue systématique, co-bénéfices sanitaires des politiques climatiques, atténuation du changement climatique
